# CC-01 (chidamide plus celecoxib) modifies the tumor immune microenvironment and reduces tumor progression combined with immune checkpoint inhibitor

**DOI:** 10.1038/s41598-022-05055-8

**Published:** 2022-01-20

**Authors:** Jia-Shiong Chen, Cheng-Han Chou, Yi-Hong Wu, Mu-Hsuan Yang, Sz-Hao Chu, Ye-Su Chao, Chia-Nan Chen

**Affiliations:** 1 New Drug Research and Development Center, Great Novel Therapeutics Biotech & Medicals Corporation (GNTbm), Taipei, Taiwan; 2 Department of Biology, Great Novel Therapeutics Biotech & Medicals Corporation (GNTbm), Taipei, Taiwan; 3 Department of Chemistry, Great Novel Therapeutics Biotech & Medicals Corporation (GNTbm), Taipei, Taiwan

**Keywords:** Cancer, Drug discovery, Immunology, Medical research

## Abstract

Immune checkpoint inhibitors (ICIs) have shown clinical benefit in solid tumors, with modest rates of clinical response. Hence, improved therapeutic approaches need to be investigated. Herein, we assessed a combination of chidamide plus celecoxib (called CC-01) combined with programmed cell death protein 1 (PD-1) blockade in a CT26 model as potent tumor microenvironment (TME) regulator. The antitumor activity was assessed by measuring tumor size, overall response rate, and survival rate. Immune profiling of tumor-infiltrating lymphocytes was performed by flow cytometry. Tumor tissues were assessed by chip assay to predict the possible pathway. Tumor size was significantly reduced in mice treated with CC-01 combined with or without anti-PD-1 antibody, however the triple combination therapy consistently demonstrated that it significantly increased both the ORR and survival rate in term of clinical applications. In the combination group, immune landscape profiling revealed decreased populations of immunosuppressive regulatory T cells, myeloid-derived suppressor cells, and tumor-associated macrophages. Analysis of the mouse tumor chip data using Gene Ontology enrichment analysis of biological processes revealed that the triple combination upregulated genes associated with responses to interferon-gamma. Our results demonstrated that CC-01 possessed potent TME regulatory properties, augmenting the antitumor effect when combined with ICIs. This antitumor effect was achieved by altering the immune landscape in TILs (tumor-infiltrating lymphocytes) and was associated with immune cell activation in the TME. Furthermore, CC-01 demonstrated potent anticancer immune response activity, mainly reducing the number and function of several immunosuppressive cells. The combination of CC-01 with an ICI will further enhance the anticancer effect and boost the immune response rate. Collectively, our results support the clinical evaluation of CC-01 in combination with ICIs in several advanced cancers.

## Introduction

Tumor immune evasion is a crucial mechanism that triggers tumor progression. It is generally accepted that CD8^+^ T cells are directly involved in antitumor cytotoxic responses, the role of CD4^+^ T cells in regulating antitumor immunity has been associated with their assistance in priming of CD8^+^ T cells, through activation of antigen-presenting cells (APC) and an increase in antigen presentation by major histocompatibility complex class I (MHC-I) molecules, via the secretion of cytokines such as interferon (IFN)-γ^1^. Targeting immune checkpoints by blocking monoclonal antibodies, including anti-programmed cell death protein 1 (PD-1) or anti-PD-L1 antibody, have provided clinical benefits for patients with advanced metastatic melanoma, non-small cell lung cancer (NSCLC), renal cell carcinoma (RCC), and several other cancers^2^. However, T cell activation remains inadequate in killing most tumors, resulting in only a small fraction of patients responding to these therapies^3^. Therefore, it is imperative to investigate effective combination regimens to successfully treat patients using immune checkpoint inhibitors (ICIs).

It is well established that tumor immune evasion involves regulatory T cells (FoxP3^+^Tregs) and myeloid-derived suppressor cells (MDSCs) to suppress tumor-specific immune responses and establish an immunosuppressive tumor microenvironment (TME)^4^. Tumor-infiltrating FoxP3^+^ Tregs play a direct role in promoting immune evasion by upregulating markers associated with activation and enhanced suppressive activity, including cytotoxic T-lymphocyte-associated protein 4 (CTLA-4), PD-1, and CD25^5,6^. The accumulation of a higher Treg:T effector cell ratio within tumor tissues is reportedly associated with poor prognosis in several cancers, including ovarian^7^ and lung^8^ cancers. MDSCs consist of two major subpopulations, monocytic MDSCs (M-MDSCs) and polymorphonuclear MDSC (PMN-MDSCs)^9^. In recent years, increasing numbers of preclinical and clinical studies have been performed to target MDSCs with beneficial effects, resulting in tumor growth inhibition and survival prolongation. The tumor-resident macrophages, as well as MDSCs, can differentiate into tumor-associated macrophages (TAMs)^10,11^. These tissue-resident macrophages undergo changes in phenotype and function during carcinogenesis, and proliferation seems key to maintain TAMs derived from tissue-resident macrophages^12^. In the early-stage cancer, the dominant TAM phenotype is reportedly tumor-promoting (M2 macrophages), as opposed to tumoricidal (M1 macrophages)^13^. Colony-stimulating factor-1 (CSF-1) receptor (CSF1R)-mediated signaling is crucial for macrophage differentiation, and the intratumoral presence of CSF1R^+^ macrophages correlates with poor survival in various tumor types^14^.

Preclinical studies have revealed that histone deacetylase (HDAC) inhibitors modulate the activation state of the APCs to effectively prime naive Ag-specific CD4^+^ T cells and restore the responsiveness of tolerant T cells isolated from tumor-bearing mice^15^. HDAC inhibitors profoundly impact T-cell development, the maintenance of the naive T-cell compartment, and important T-cell activation pathways, all of which possibly impact antitumor T-cell responses^16^. Benzamide-based class I HDAC inhibitors include well known compounds such as entinostat (MS-275), mocetinostat (MGCD0103), chidamide (tucidinostat/HBI-8000). Currently, entinostat and chidamide are under development for clinical trials as both single-agent and combination therapies^17^. Reportedly, chidamide selectively inhibits the activity of HDACs 1, 2, 3, and 10, demonstrating its anticancer functions as a genuine epigenetic modulator via the following mechanisms: induction of growth arrest and apoptosis in the blood and lymphoid-derived tumor cells; the reversal of epithelial-mesenchymal transitions and drug resistance in tumor cells; importantly, enhancement of natural killer (NK)-cell and antigen-specific CD8^+^ cytotoxic T-lymphocyte (CTL) mediated cellular antitumor immunity^18–22^. A recent study has evaluated nivolumab in association with HBI-8000 (chidamide/tucidinostat) for immunotherapy^23^. Prostaglandin E2 (PGE2) is a critical product of cyclooxygenase 2 (COX-2), which is overexpressed in most human cancers, affects tumor progression and immunosuppression, and stimulates arginase-1 (ARG-1) and nitric oxide synthase (NOS)-2 secretion from MDSCs^24^. PGE2 can induce tumor growth and suppress immune functions by promoting the development of cluster of differentiation CD4^+^ and CD25^+^ regulatory T cells (Tregs) in the TME^25^. Therefore, the main objective of this study was to develop a new combination chidamide + celecoxib regimen (called CC-01) to improve tumor clearance rates of immune checkpoint inhibitors by controlling the TME, and further improve survival rates.

## Methods

### Materials

Chidamide-API and chidamide-K30 (pre-formulation) were provided by GNT Biotech & Medicals Co. Ltd (Taipei, Taiwan). Celecoxib-API was purchased from Aarti Drugs Ltd. (India). Celecoxib capsules (Celebrex^®^, 200 mg) were purchased from Pfizer (Pfizer Canada Inc.). The following antibodies and reagents were used for animal experiments: mouse anti-PD-1 (CD279) monoclonal antibody (RMP1-14; Bio X Cell), mouse anti-PD-L1 (B7-H1) monoclonal antibody mouse (BE0101; Bio X Cell), anti-CTLA-4 (CD152) monoclonal antibody (BE0164; Bio X Cell), and rat anti-IgG2a isotype control monoclonal antibody (2A3; Bio X Cell). Distilled water was purified using the Milli-Q distillation system (Merck Millipore^®^, France).

### Cell lines and cell viability assay

CT26 cells (CRL-2638; murine colorectal adenocarcinoma) were purchased from ATCC. Two different cell lines, including the human breast cancer cell line, MDA-MB-231 (6 × 10^3^), and the human breast epithelial cell line, M10 (6 × 10^3^), were seeded in 96 well of plates. Cell lines were obtained from Bioresource Collection and Research Center, BCRC, Taiwan. All cell lines were treated with chidamide and celecoxib, with doses ranging from 0.8125 to 50 μM, and then incubated at 37 °C under 5% CO_2_ for 72 h. After 72 h, the MTT assay (Cayman™) was used to determine cellular viability. The MDA-MB-231 cell line was maintained in Dulbecco’s Modified Eagle Medium (DMEM)/F12 supplemented with 10% fetal bovine serum (FBS) and 0.2% antibiotic (MycoZap™, Plus-CL). The M10 cell line was maintained in MEM Alpha (Gibco™) supplemented with 10% FBS and 0.2% antibiotic (MycoZap™, Plus-CL). The CT26 tumor cell line was cultured in McCoy’s 5A, supplemented with 10% (vol/vol) FBS at 37 °C and 5% CO_2_. Gibco RPMI 1640 and DMEM with l-glutamine were purchased from Invitrogen Life Technologies. HyClone FBS was purchased from Thermo Fisher Scientific.

### Animal models

The animal study was approved and monitored by the Taipei Medical University Institutional Animal Care and Use Committee (TMU IACUC, NO: LAC-2018-0340). All experiments, 6–8-week-old male wild BALB/C and NU-*Foxn1*^nu^ nude mice (BioLASCO Taiwan) were bred in Taipei Medical University Laboratory Animal Center under specifc-pathogen-free conditions with fresh water and rodent diet available at all times. All mice were kept in microisolator cages under a 12-h day/night cycle and were carried out under protocols that complied with the Institutional Animal Care and Use Committee Guidelines for Ethical Conduct in the Care. This study is reported in accordance with ARRIVE guidelines. CT26 (1 × 10^7^) cancer cells were subcutaneously inoculated into the right flank of each mouse, and tumors were allowed to grow for 9–11 days (tumor size approximately 200–300 mm^3^) before randomization and treatment. CT26-bearing mice were intraperitoneally administered 2.5 mg/kg of anti-IgG (Lot#65481701), anti-PD-1 (Lot#640517M1 and Lot#717918D1), anti-PD-L1 (Lot#720619F1), or anti-CTLA-4 (Lot#702418A2B) antibody on days 11, 14, 17, 20, 23, and 26 post-tumor implantation. Anti-CD8 (Lot#BE0061) or anti-CD4 (Lot#BE0003-1) antibody was administered 200 μg/mouse 2 days before drug treatment and thereafter on days 13, 16, and 19 post-tumor implantation. All antibodies were diluted to appropriate concentrations in 100 μL of sterile phosphate-buffered saline (PBS; pH 7.4) (Invitrogen Life Technologies). Chidamide-K30 and celecoxib (capsule/Celebrex^®^, 200 mg) were orally administrated on day 11 (dependent tumor size) post-tumor implantation. Chidamide-K30 was orally administered to treat tumor-bearing mice at doses of 12.5, 25, and 50 mg/kg, daily from days 11 to 26. Daily celecoxib treatment (capsule/Celebrex^®^, 200 mg) was performed at doses of 12.5, 25, and 50 mg/kg from days 11 to 26. The anticancer activity was measured from the start of treatment until the tumor volume reached 3000 mm^3^. Tumor volume was calculated as length × width^2^ × 0.5. To evaluate efficacy, the following grading was defined: complete response (CR), less than 0.5-fold tumor growth; partial response (PR), equal to or greater than 0.5-fold, equal to or less than twofold tumor growth; stable disease (SD), greater than twofold and less than fivefold tumor growth; progression disease (PD), equal to or greater than fivefold tumor growth, as compared to baseline. The overall response rate (ORR) is the percentage of CR + PR.

### Survival rate in animal models

Antibody or drug administration was performed from days 11 to 25 or 26. Tumor growth continued in tumor-bearing mice, and tumor volume was measured once every 3 or 4 days (twice/week). Tumor-bearing mice were regarded as dead when the tumor volume reached 3000 mm^3^. Use carbon dioxide to sacrifice mice when reaching 3000 mm^3^. All treatment groups were recorded and analyzed.

### Flow cytometry

The following antibodies and reagents were used for flow cytometry.

CD8a PerCP-Cy5.5 (53-6.7; BioLegend), CD4 PE (GK 1.5; BioLegend), CD4 APC (GK 1.5; BioLegend), CD25 PerCP-Cy5.5 (PC61; BioLegend), Foxp3 PE (MF14; BioLegend), CD3 APC (17A2; BioLegend), CD11b APC (M1/70; BioLegend), Ly-6C PerCP-Cy5.5 (HK 1.4; BioLegend), Ly-6G PE (1A8; BioLegend), MHC-ll-PE (BM8; BioLegend), CD45 FITC (30-F11; BioLegend), PD-1 PE (REA 802; Miltenyi Biotec, Germany), TIM-3 APC (REA 602; Miltenyi Biotec, Germany), LAG-3 APC (C9B7W; BioLegend), GzmB PE (REA 226; Miltenyi Biotec, Germany), Ki67 PE (REA 183; Miltenyi Biotec, Germany), INF-γAPC (REA 683; Miltenyi Biotec, Germany). Flow cytometry was performed using the FACS Caliber flow cytometer (BD Biosciences), and data were analyzed with FACSDiva™ software (BD Biosciences). To assess the levels of circulating cell populations, mouse blood samples were collected 12 days after chidamide + celecoxib treatment initiation, with or without anti-PD-1 antibody. In total, 150 µL of blood was collected in a K2EDTA BD Microtainer (BD Biosciences) from the right or left facial vein. Red blood cells (RBCs) from anticoagulated blood samples were immediately lysed using 2 mL of 1× RBC lysis buffer (Qiagen, Valencia, CA) for 10 min, and samples were washed twice in ice-cold PBS (BD Biosciences). The samples were stained with the appropriate antibodies. For analysis, we used previously established phenotypic criteria of these cells, with CD45^+^/CD11b^+^/Ly6G^+^/Ly6C^-^ cells (PMN-MDSC) and CD45^+^/CD11b^+^/Ly6G^−^/ Ly6C^+^ (M-MDSC), CD45^+^/CD4^+^/CD25^+^/Foxp^+^ cells (Treg), and CD45^+^/CD3^+^/CD4^+^ cells (CD4) and CD45^+^/CD3^+^/CD8^+^ (CD8) T cell, CD45^+^/CD11b^+^/Ly6C^+^/MHCll^+^ (TAM), CD45^+^/CD8^+^/TIM-3^+^ cells (CD8^+^TIM-3^+^), CD45^+^/CD8^+^/LAG-3^+^ cells (CD8^+^LAG-3^+^), CD45^+^/CD8^+^/PD-1^+^ cells (CD8^+^PD-1^+^), CD45^+^/CD8^+^/Ki67^+^ cells (CD8^+^Ki67^+^), CD45^+^/CD8^+^/GzmB^+^ cells (CD8^+^GzmB^+^), CD45^+^/CD8^+^/INF-γ^+^ cells (CD8^+^INF-γ^+^), and total mononuclear cells used as a common denominator. The cells were then fixed and permeabilized with BD Cytofix/Cytoperm (BD Biosciences) and then stained with antibody against IFN-γ, Ki67, Foxp, or GzmB Ab. The cells were sorted with a BD Accuri C6 and the data were analyzed by using BD Accuri C6 software.

To assess the level of tumor infiltrated lymphocytes, intratumoral CD8^+^, CD4^+^, Treg, PMN-MDSC, M-MDSC, and TAM populations were analyzed. The tumor infiltrated lymphocytes were first purified from mice tumor samples excised on day 12 after chidamide + celecoxib treatment initiation, with or without anti-PD-1 antibody. Briefly, primary tumor tissues were harvested, weighed, and minced to fine fragments. To each sample, collagenase IV (Sigma-Aldrich) at 1 mg/mL in HBSS (Invitrogen Life Technologies) was added at a ratio of 1 mL per 200 mg of tumor tissue. Next, samples were incubated on an end-over-end shaker for 150 min at 37 °C. The resulting tissue homogenates were filtered using a 0.4-μm filter, washed three times in PBS (BD Biosciences), and separated by Percoll gradient to isolate mononuclear cells; 1 × 10^6^ cells per sample were used for antibody labeling.

### RNA extraction, cDNA microarray experiment, and analysis

RNA extraction and microarray hybridization experiments as previously description^26^. Approximately 100 mg of frozen tissue was homogenized with Precellys 24^®^ equipment (Carlsbad, California, USA). Next, the supernatant was used to purify total RNA using the RNeasy Mini kit (Qiagen, Venlo, the Netherlands) according to the manufacturer’s protocol. The quantity and purity of RNA samples were assessed with NanoDrop™ ND-1000 (Thermo Scientific, Wilmington, Delaware, USA). RNA integrity was controlled with the Agilent Bioanalyzer 2100 (Agilent Technologies, Palo Alto, California, USA). Only RNA samples with optical density (OD)_260/280_ > 1.8 and RIN (RNA Integrity Number) > 6 were further processed. The labeled cRNA from 4 samples was synthesized using the two-color microarray-based gene expression analysis Low Input Quick Amp-Labeling Kit (Agilent Technologies, Santa Clara, USA) following the manufacturer’s standard protocol. Briefly, 0.2 μg of total RNA was amplified by a Low Input Quick Amp Labeling kit (Agilent Technologies, USA) and labeled with Cy3 (CyDye, Agilent Technologies, USA) during the in vitro transcription process. Then, 0.6 μg of Cy3-labeled cRNA was fragmented to an average size of approximately 50–100 nucleotides by incubation with the fragmentation buffer at 60 °C for 30 min. Then, correspondingly fragmented labeled cRNA was pooled and hybridized with Agilent SurePrint Microarray (Agilent Technologies, USA) at 65 °C for 17 h. After washing and drying by nitrogen gun blowing, microarrays were scanned with an Agilent microarray scanner (Agilent Technologies, USA) at 535 nm for Cy3. Scanned images are analyzed by feature extraction 10.7.3.1 software (Agilent Technologies, USA), an image analysis and normalization software used to quantify signal and background intensities for each feature. Raw signal data were normalized by quantile normalization for determining differentially expressed genes. For the functional assay, we presented an enrichment test for differentially expressed genes (for most model organisms); Welgen Biotech used clusterProfiler for the enrichment test for Gene Ontology (GO) and pathway [Kyoto Encyclopedia of Genes and Genomes (KEGG)]. GO terms with statistically significant (p < 0.05) and p-value adjusted by FDR for significance discovering. For microarray analysis, only the annotated genes were considered. Data were analyzed by comparing treated samples against the IgG sample.

### RNA isolation and real-time reverse transcription-polymerase chain reaction (RT-PCR)

Total RNA was isolated and Q-PCR reaction as previously described^27^. Total RNA was isolated using TRIZOL reagent (Invitrogen, Carlsbad, CA, USA) and the first-strand cDNA was synthesized from 2 μg of total RNA using the cDNA Reverse Transcription Kit with RNase Inhibitor (Invitrogen) according to the manufacturer’s instructions. Real-time RT-PCR was performed using SYBR Green PCR Master Mix (Applied Biosystems). The reactions were performed using the 7500 Fast Real-Time PCR system (Applied Biosystems, Carlsbad, CA), and cycling conditions were as follows: initial denaturation at 95 °C for 2 min, and then 40 cycles of denaturation at 95 °C for 15 s, annealing at 60 °C for 30 s, and extension at 72 °C for 1 min. Melting curve analysis was performed to assess the amplification of the desired gene product. Data were analyzed with the 2^−ΔΔCt^ method, using GAPDH as the internal control. The following primers were designed base on Primer BLAST performs and utilized for PCR: NOS-2 Forward: CAGGTGCCCTCTAGCACTTC, NOS-2 Reverse: CTGAGGCGACAGAAGGTAGG; interleukin (IL)-4Rα Forward: GTGCTTCTGAGGGAGAGTGG, IL-4Rα Reverse: TCAGAGGGAGGCTAGTGCAT; IL-6 Forward: CCGGAGAGGAGACTTCACAG, IL-6 Reverse: TCCACGATTTCCCAGAGAAC; IL-10 Forward: TCAGAGCTCCTGGAACTGGT, IL-10 Reverse: CACCTGTGTCAACCCTTCCT; chemokine ligand 8 (CCL8) Forward: TTGTACACTGAGGGGCTTCC, CCL8 Reverse: CAAGAACTCGCTGTCCATCA; C-X-C motif chemokine ligand 10 (CXCL10) Forward: GGATGGCTGTCCTAGCTCTG, CXCL10 Reverse: ATAACCCCTTGGGAAGATGG; CSF1 Forward: CCTGCCACCTGTATGACCTT, CSF1 Reverse: TCTTGGGCAGGTCTGAGAGT; transforming growth factor (TGF)-β Forward: TTTCCCATGGAGAGATGAGG, TGF-β Reverse: CTGGCCATTTCACCAGTTTT, CSF2 Forward: TCCTAGAATGGGCAGACACC, CSF2 Reverse: CTGAGGCATCTCCTCACCTC; INF*-*r Forward: TGGGCTTTGATGATGAATGA, INF*-*r Reverse: AGCGGGAGGCTAGTTAGAGG.

### Statistics

All statistical analyses were performed using GraphPad Prism 5.0 (GraphPad Software, Inc., La Jolla, CA, USA). Primary tumor growth curves, flow cytometric analyses, and in vitro assays were first analyzed with one-way ANOVA, and individual groups were compared using Tukey’s Multiple Comparison Test. Kaplan–Meier survival curves were analyzed with a log-rank test. Gene expression level in CT26 tumors was analyzed by unpaired Student’s t-test.

### Ethics statement

This study was carried out in accordance with the recommendations of the Medical Animal Care and Welfare Committee of Taipei Medical University. The protocol was approved by the Medical Animal Care and Welfare Committee of Taipei Medical University.

## Results

### Chidamide enhances the antitumor activity of anti-PD-1 antibody in CT26-bearing mice

Reportedly, immune checkpoint-blocking antibodies have revealed poor anticancer response rate, which can be enhanced in combination with epigenetic agents^28^. To test this hypothesis, we treated animals bearing CT26 tumors (~ 200 mm^3^) with class I HDAC inhibitors, entinostat (MS-275) (20 mg/kg) or chidamide (25 mg/kg), as well as anti-PD-1 antibody (10 mg/kg). Although repeated treatment with the anti-PD-1 antibody or CD (chidamide) as a single agent retarded tumor growth, tumor eradication was not observed (SFig. 1A,B). The combination of anti-PD-1 antibody with entinostat (as positive control) resulted in significant suppression of tumor growth, but no eradication of primary tumors (complete response, CR) in 7 mice, extending survival to 70% at day 42 after tumor implantation when compared with anti-PD-1 antibody (SFig. 1C,D). Similarly, the combined anti-PD-1 antibody + chidamide treatment regimen showed synergistic activity, with primary tumor eradication in 1 out of 5 mice and 40% survival at day 42 after tumor implantation (SFig. 1D). Next, we planned to further improve the response rate to meet clinical needs.

### Treatment of mice with triple combination chidamide + celecoxib + anti-PD-1 antibody

**Figure 1:**
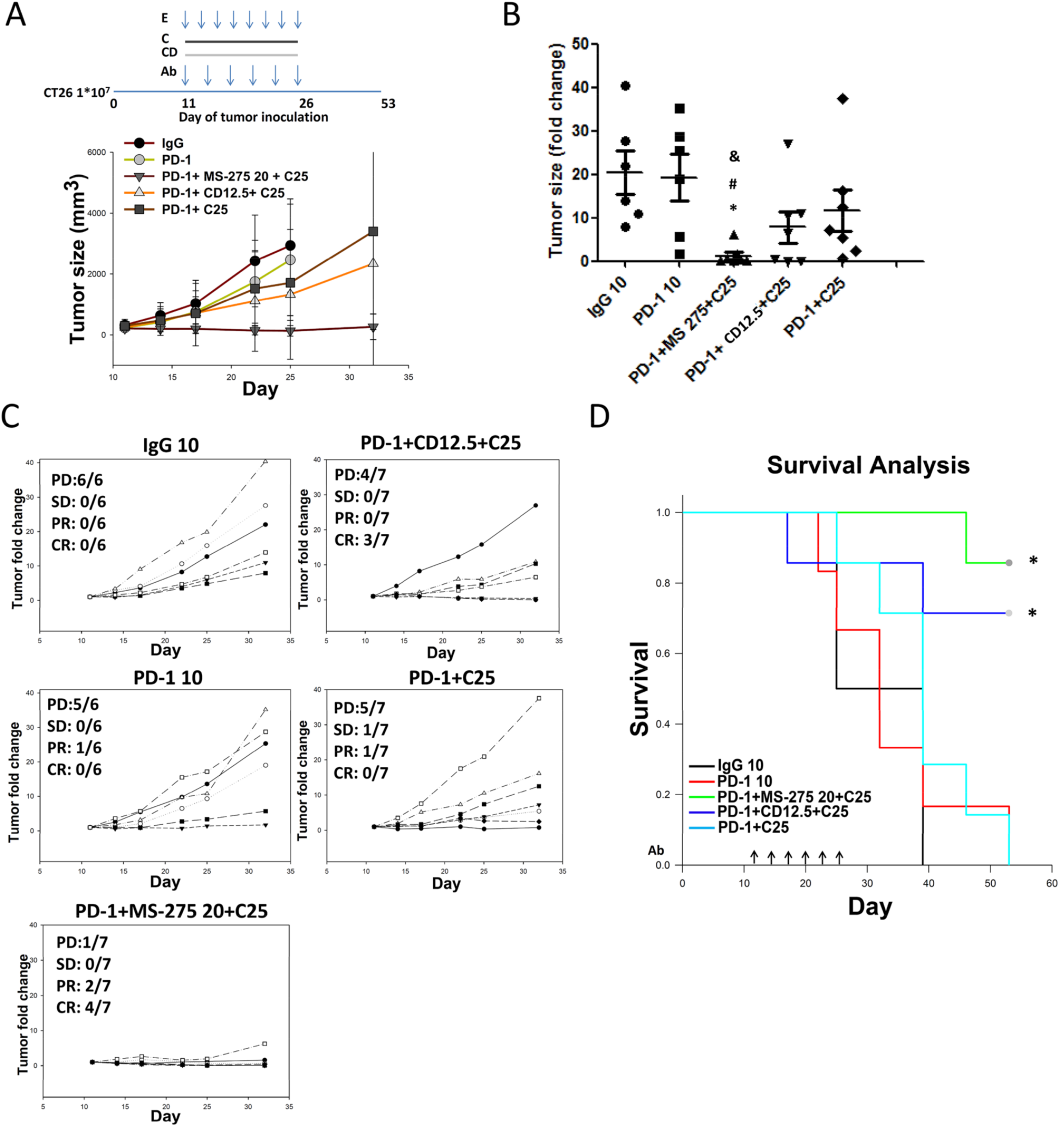
Chidamide + celecoxib enhances anti-PD-1-induced immunotherapy in an allograft CT26 mouse model. (**A**) CT26 tumor-bearing mice were orally administered a chidamide-k30 solution (CD, 25 mg/kg) once daily from days 11 to 26 (Day 11 mean TV, 200–250 mm^3^), entinostat (E, 20 mg/kg) once every 2 days from days 11 to 26, and celecoxib (C, 25 mg/kg) once daily from days 11 to 26. Arrows indicate the time points at which mice were treated with the anti-PD-1 antibody. The mice were treated with anti-PD-1 antibody (10 mg/kg, i.p.) once every 3 days on days 11, 14, 17, 20, 23, and 26. Entinostat (MS-275), as a positive control, was administered with anti-PD-1 following the indicated schedules. (**B**) Endpoint tumor size is presented as tumor volume (TV) and fold change. Results are shown as mean ± standard error of the mean (SEM). *p < 0.05 vs. anti-IgG; ^#^p < 0.05 vs. anti-PD-1; ^&^p < 0.05 vs. CD25. (**C**) Individual tumor growth graphs for CT26 tumor-bearing mice (n = 6–7). Number of tumor-free mice per total number of mice is shown at the top left corner of each panel. (**D**) Survival study of all the treatment groups.

PGE2 is known to promote tumor progression in the TME^25^. To increase the antitumor response rate, we introduced celecoxib (a selective COX-2 inhibitor) in this treatment regimen, evaluating this hypothesis with a triple combination of anti-PD-1 Ab + chidamide + celecoxib in CT26-bearing mice. We treated animals bearing CT26 tumors (240 ± 25 mm^3^ on day 11) with anti-PD-1 Ab (10 mg/kg), chidamide (12.5 mg/kg) or entinostat (20 mg/kg), and celecoxib (25 mg/kg). The tumors responded to the anti-PD-1 Ab + entinostat + celecoxib regimen remarkably well (Fig. 1A), eradicating primary tumors in 4 out of 7 mice, with 85% survival at day 53 after tumor implantation (Fig. 1B–D). Similarly, in response to combination anti-PD-1 Ab + chidamide (12.5 mg/kg) + celecoxib (25 mg/kg) treatment, mice with CT26 tumors revealed reduced progression of all primary tumors, eradication of primary tumors in 3 out of 7 mice, and 71% survival at day 53 after tumor implantation (Fig. 1B–D). Conversely, the anticancer activity and primary tumor eradication ability of the regimen lacking chidamide were inadequate (anti-PD-1 antibody + celecoxib 25 mg/kg). Chidamide demonstrated its most important function in the combination treatment regimen composed of PD-1 Ab + chidamide + celecoxib (Fig. 1B–D).

Next, we investigated the optimal dosage regimen and confirmed whether the addition of celecoxib to the anti-PD-1 antibody plus chidamide would provide additional benefits. According to our unpublished data, we have tried chidamide 50 mg/kg and celecoxib 50 mg/kg. But we don’t know whether the drug concentration is related to the efficacy under the condition of combined antibody. First, we evaluated the anti-PD-1 antibody dosage at 2.5, 5 and 10 mg/kg to analyze whether a low antibody dosage could demonstrate similar results in the combination regimen. The results showed no significant differences in these three groups (SFig. 2). Second, we evaluated mice bearing CT26 tumors treated with anti-PD-1 antibody (2.5 mg/kg), with increasing doses of chidamide (12.5, 25, and 50 mg/kg) and celecoxib (12.5, 25, and 50 mg/kg). The anti-PD-1 antibody + chidamide (50 mg/kg) + celecoxib (50 mg/kg) regimen demonstrated the best antitumor response, with primary tumor eradication in 3 out of 4 mice and 50% survival at day 58 after tumor implantation (SFig. 3). Based on the above findings, we selected the low dose of the anti-PD-1 antibody for further animal experiments. Conversely, we observed that the chidamide (50 mg/kg) + celecoxib (50 mg/kg) regimen was an optimal combination for further investigation.

**Figure 2:**
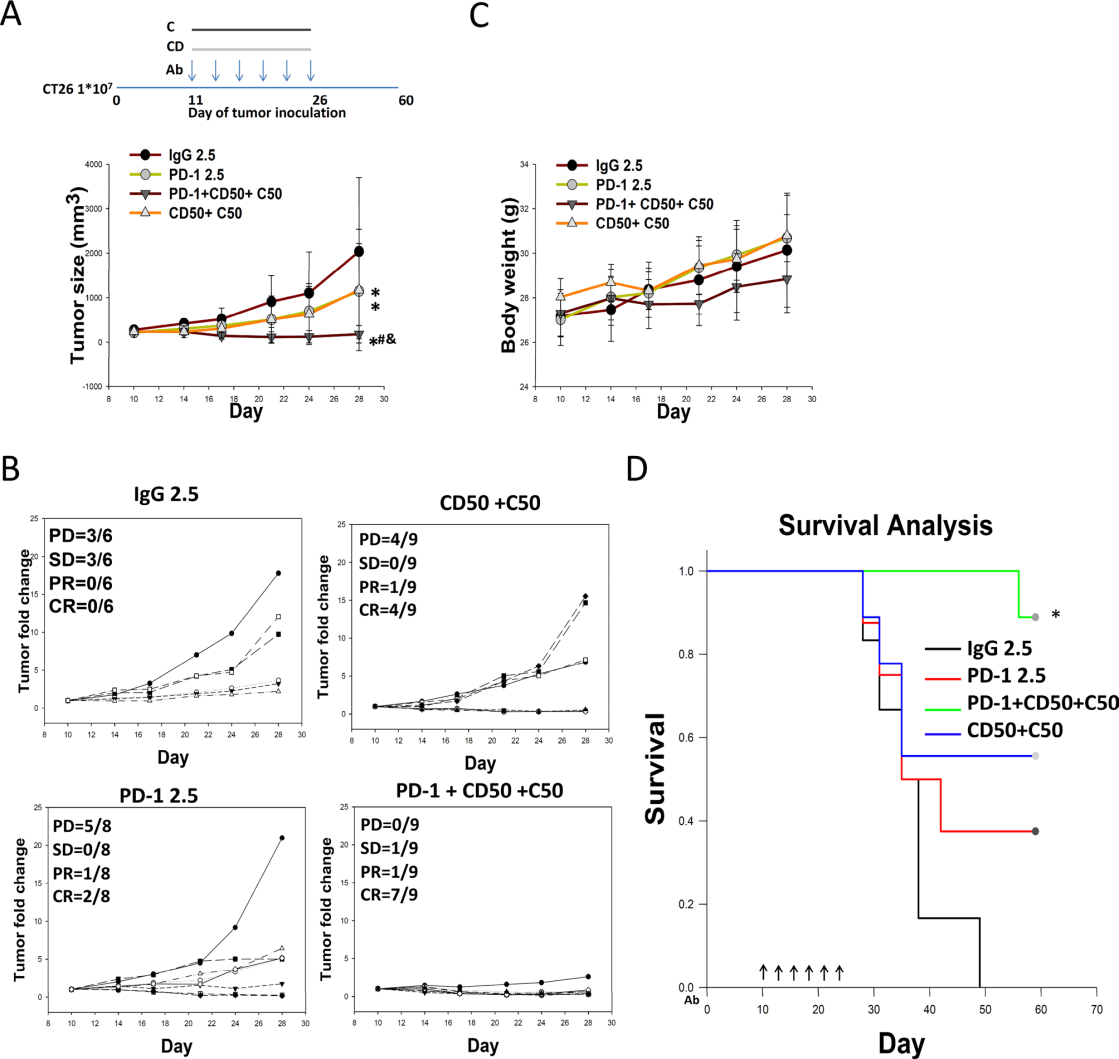
Chidamide + celecoxib suppress tumor growth and enhance anti-PD-1-induced immunotherapy in an allograft CT26 mouse model. (**A**) CT26 tumor-bearing mice were orally administered a chidamide-k30 solution (CD, 50 mg/kg) once daily from days 10 to 25 (Day 10 mean TV, 240 ± 25 mm^3^), and celecoxib (C, 50 mg/kg) once daily from days 10 to 25. Arrows indicate the time points at which mice were treated with the anti-PD-1 antibody. The mice were treated with anti-PD-1 antibody (2.5 mg/kg, i.p.) once every 3 days on days 10, 13, 16, 19, 22, and 25. Endpoint tumor size is presented as fold change. Results are shown as mean ± standard deviation (SD). *p < 0.05 vs. anti-IgG; ^#^p < 0.05 vs. anti-PD-1; ^&^p < 0.05 vs. CD50 + C50. (**B**) Individual tumor growth graphs for CT26 tumor-bearing mice (n = 6–9). Number of tumor-free mice per total number of mice is shown at the top left corner of each panel. (**C**) Body weight of mice following each treatment was recorded. (**D**) Survival study of all the treatment groups.

To further confirm the antitumor activities of these regimens chidamide plus celecoxib with or without of anti-PD-1 Ab, we increased the animal number and repeated these treatment regimens. Our findings showed that tumors responded to anti-PD-1 Ab (2.5 mg/kg), with primary tumor eradication in 2 out of 8 mice, and 37.5% survival at day 59 after tumor implantation (Fig. 2A,B,D). In the chidamide (50 mg/kg) + celecoxib (50 mg/kg) regimen, tumor growth was significantly suppressed, with primary tumor eradication in 4 out of 9 mice, and 55.5% survival at day 59 after tumor implantation (Fig. 2A,B,D). The anti-PD-1 antibody + chidamide (50 mg/kg) + celecoxib (50 mg/kg) regimen resulted in significant suppression of tumor growth, with primary tumor eradication in 7 out of 9 mice, and 88.9% survival at day 59 after tumor implantation (Fig. 2A,B,D). Additionally, the triple combination regimen with anti-PD-1 antibody + chidamide (50 mg/kg) + celecoxib (50 mg/kg) did not increase toxicity by causing body weight loss (Fig. 2C).

**Figure 3:**
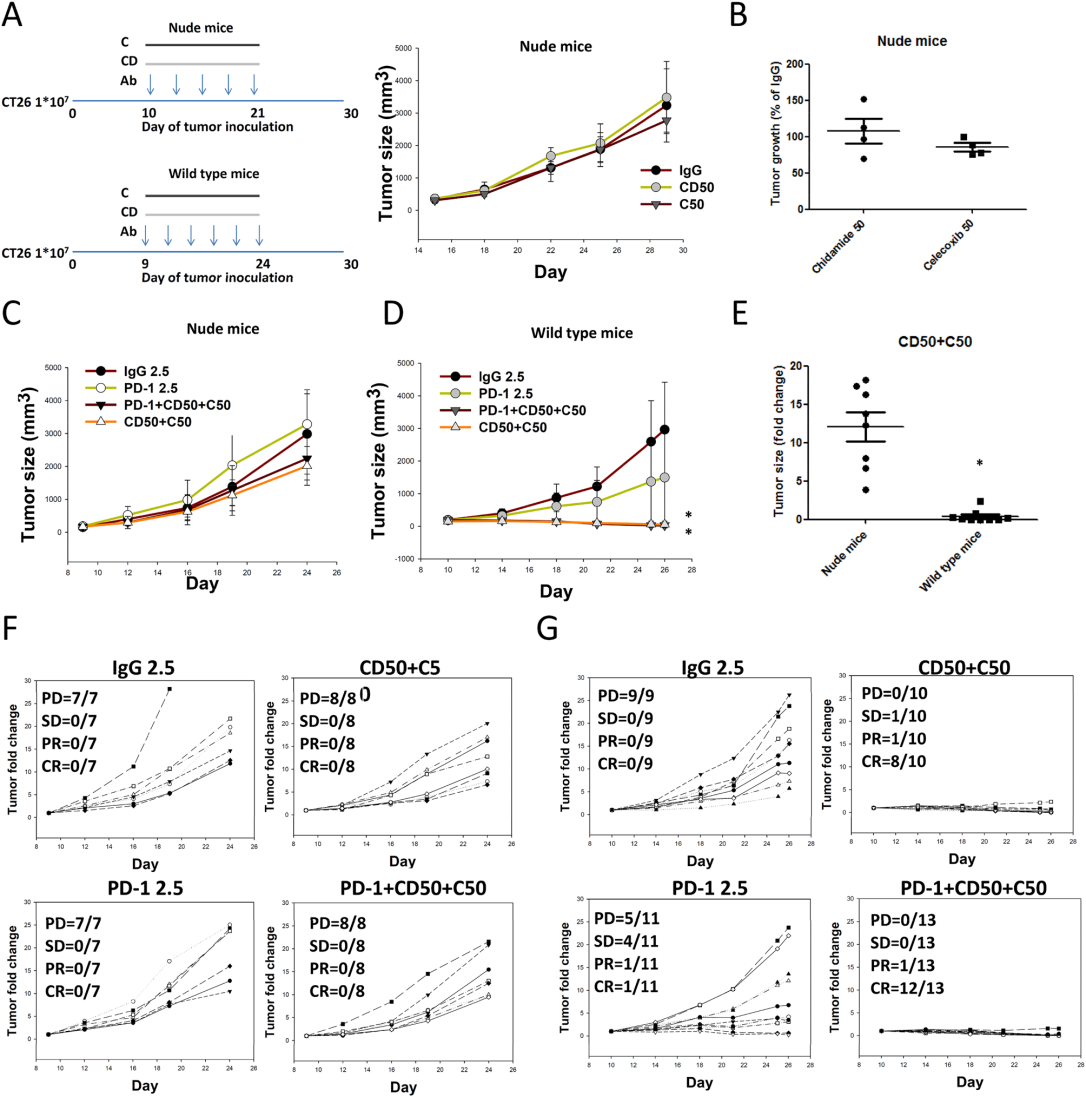
Chidamide + celecoxib demonstrate anticancer activity by immune activation and enhance anti-PD-1-induced immunotherapy in a wild type and immunodeficient nude mice allograft CT26 mouse model. (**A**) CT26 tumor-bearing nude mice were orally administered a chidamide-k30 solution (CD, 50 mg/kg) once daily from days 10 to 21 (Day 15 mean TV, 174 ± 39 mm^3^), and celecoxib (C, 50 mg/kg) once daily from days 15 to 29. Endpoint tumor size is presented as tumor volume (TV; mm^3^). Results are shown as mean ± standard deviation (SD). *p < 0.05 vs. anti-IgG; (n = 4). (**B**) Tumor growth suppression % graphs for CT26 tumor-bearing nude mice. (**C**) CT26 tumor-bearing nude mice were orally administered a chidamide-k30 solution (CD, 50 mg/kg) once daily from days 9 to 21 (Day 9 mean TV, 191 ± 10 mm^3^), and celecoxib (C, 50 mg/kg) once daily from days 9 to 24. The mice were treated with anti-PD-1 antibody (2.5 mg/kg, i.p.) once every 3 days on days 9, 12, 15, 18, 21, and 24. Endpoint tumor size is presented as tumor volume (TV; mm^3^). Results are shown as mean ± standard error of the mean (SEM). *p < 0.05 vs. anti-IgG; (n = 7–8). (**D**) CT26 tumor-bearing wild type mice were orally administered a chidamide-k30 solution (CD, 50 mg/kg) once daily from days 8 to 23 (Day 10 mean tumor volume (TV), 150–190 mm^3^), and celecoxib (C, 50 mg/kg) once daily from days 8 to 23. The mice were treated with anti-PD-1 antibody (2.5 mg/kg, i.p.) once every 3 days on days 8, 11, 14, 17, 20, and 23. Endpoint tumor size is presented as TV (mm^3^). Results are shown as mean ± standard error of the mean (SEM). *p < 0.05 vs. anti-IgG; (wild type mice n = 8). (**E**) Tumor growth suppression % graphs for CT26 tumor-bearing nude vs. wild type mice (n = 8 and 20). (**F**) Individual tumor growth graphs for CT26 tumor-bearing nude mice (n = 7–8). Number of tumor-free mice per total number of mice is shown at the top left corner of each panel. (**G**) Individual tumor growth graphs for CT26 tumor-bearing wild type mice (n = 9–13). Number of tumor-free mice per total number of mice is shown at the top left corner of each panel.

To further assess the role of immune cells in the anti-tumorigenic response, we compared the response to the triple combination regimen in immunodeficient nude mice. Cg-Foxn1^nu^/CrlBltw mice lack a thymus and cannot produce T cells, thus causing immunodeficiency. In the nude mice, monotherapy with chidamide (50 mg/kg) or celecoxib (50 mg/kg) failed to reduce tumor growth (Fig. 3A–C). However, the combination chidamide (50 mg/kg) + celecoxib (50 mg/kg) regimen marginally reduced tumor growth in nude mice (tumor size 174 ± 39 mm^3^ on day 10), but significantly reduced tumor growth in wild mice (tumor size 191 ± 10 mm^3^ on day 9) (Fig. 3D,E). The combination chidamide (50 mg/kg) + celecoxib (50 mg/kg) regimen was more effective in the immunocompetent BALB/c mice and not in immunodeficient mice (Fig. 3E). Similar results were observed with the triple combination regimen chidamide + celecoxib + anti-PD-1 Ab, that is, showing slight antitumor activity even in the absence of tumor-killing T cells in nude mice (Fig. 3C,D). The efficacy results of individual mice are shown in Fig. 3F for immunodeficient mice and Fig. 3G for immunocompetent mice. To exclude the cytotoxicity effect of chidamide and celecoxib, we performed in vitro cell model to clarify this issue. The results showed that suppressed tumor proliferation, necessitating approximately 10 μM in both M10 and CT26 but > 10 μM in M231 (SFig. 4C). These results suggested that combining chidamide and celecoxib may activate immune cells such as CTL/NK cells in the TME to reduce the tumor burden.

To evaluate whether the antitumor activity of the triple combination regimen was dependent on CD4^+^ or CD8^+^ T cell activation, we compared the antitumor activity of triple combination regimen with and without the presence of CD4^+^ or CD8^+^ T cells in the CT26 mouse model by using an anti-CD4 or anti-CD8 antibody administered in wild type mice. We used flow cytometry to confirm that CD4^+^ T cell percentage was decreased as a result of cell depletion by anti-CD4 antibody administered (SFig. 5A–C). Similar result was also shown in anti-CD8 antibody depletion condition. In efficacy assay, compared with IgG treatment, treatment with anti-PD-1, chidamide + celecoxib, or the triple combination, increased ORR despite CD4^+^ T cell depletion, but not in CD8^+^ T cell-depleted conditions (SFig. 5D–G). These results suggested the antitumor activity of each treatment regimen, anti-PD-1, chidamide + celecoxib, or the triple combination, was dependent on CD8^+^ T cell activation.

### Treatment with a triple combination of class I HDAC inhibitors + COX-1/COX-2 inhibitors + ICIs to classify the anticancer mechanisms

**Figure 4:**
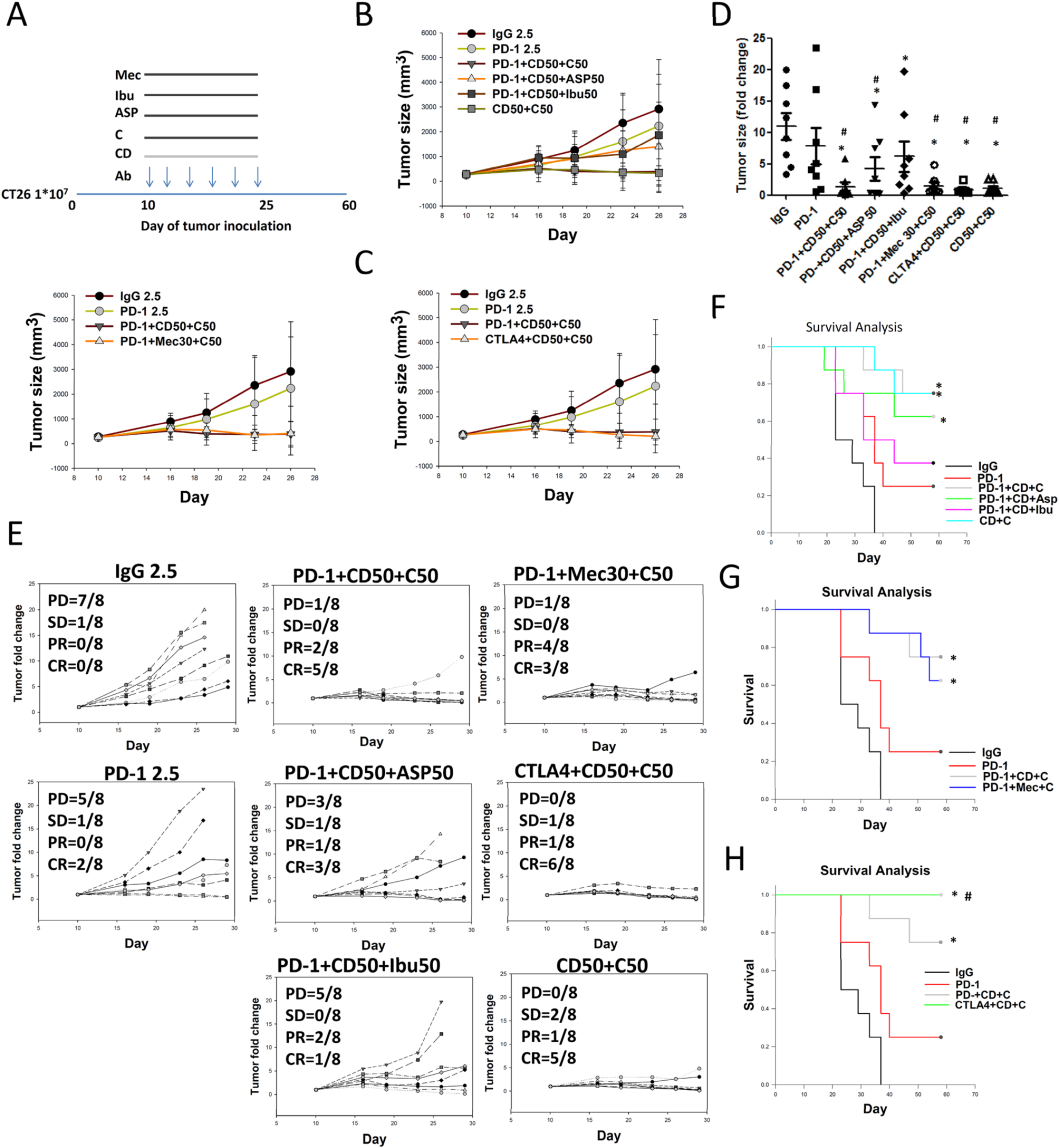
Class I HDAC inhibitors (HDACi) + COX inhibitors (COXis) enhance anti-PD-1-induced immunotherapy in an allograft CT26 mouse model. (**A–C**) CT26 tumor-bearing mice were administered class I HDACi (chidamide-k30 or mocetinostat) once daily (50 mg/kg) from days 10 to 25 (Day 10 mean tumor volume (TV), 223 ± 36 mm^3^), and COXis (celecoxib, aspirin, and ibuprofen) (50 mg/kg) once daily from days 10 to 25. Arrows indicate the time points at which mice were treated with immune checkpoint inhibitors (ICIs; anti-PD-1 and anti-CTLA4 antibody). The mice were treated with anti-PD-1 antibody (2.5 mg/kg, i.p.) once every 3 days on days 10, 13, 16, 19, 22, and 25. (**D**) Endpoint tumor size is presented as fold change. Results are shown as mean ± standard deviation (SD). *p < 0.05 vs. anti-IgG; ^#^p < 0.05 vs. anti-PD-1. (**E**) Individual tumor growth graphs for CT26 tumor-bearing mice (n = 8). Number of tumor-free mice per total number of mice is shown at the top left corner of each panel. (**F–H**) Survival study after treatment with class I HDACi + COXi + ICI combination regimen.

Given that the triple combination of anti-PD-1 Ab + chidamide + celecoxib resulted in increased anti-tumorigenic responses and improved survival in CT26-bearing mice, we next questioned whether a triple combination composed of class I HDAC inhibitors + COX-1/COX-2 inhibitors + ICIs would provide a superior anti-tumorigenic response. To evaluate this hypothesis, CT26-bearing mice (tumor size 227 ± 14 mm^3^ on day 10) treated with ICIs (anti-PD-1 or anti-CTLA-4 antibody), class I HDAC inhibitors (chidamide or mocetinostat), and COX-1/COX-2 inhibitors (celecoxib, aspirin, or ibuprofen) were evaluated. The results showed that the combination of chidamide + celecoxib significantly reduced tumor volume, with primary tumor eradication in 5 out of 8 mice, and 75% survival at day 60 after tumor implantation (Fig. 4A,D–F). The triple combination of chidamide + celecoxib + anti-PD-1 antibody significantly reduced tumor volume, with primary tumor eradication in 5 out of 8 mice, and 75% survival at day 60 after tumor implantation (Fig. 4A,D–H). A triple combination of chidamide + celecoxib + anti-CTLA-4 antibody significantly reduced tumor volume, with primary tumor eradication in 6 out of 8 mice, and 100% survival at day 60 after tumor implantation (Fig. 4C–E,H). A triple combination of mocetinostat + celecoxib + anti-PD-1 antibody significantly reduced tumor volume, with primary tumor eradication in 3 out of 8 mice, and 62.5% survival at day 60 after tumor implantation (Fig. 4B,D–F). Finally, we replaced the type of COX-1/COX-2 inhibitor in the triple combination. A triple combination of chidamide (50 mg/kg) + aspirin (50 mg/kg) + anti-PD-1 antibody significantly reduced the tumor volume, with primary tumor eradication in 3 out of 8 mice, and 37.5% survival at day 60 after tumor implantation (Fig. 4A,D–F). A triple combination of chidamide (50 mg/kg) + ibuprofen (50 mg/kg) + anti-PD-1 antibody partially reduced the tumor volume, with primary tumor eradication in 1 out of 8 mice, and 25% survival at day 60 after tumor implantation (Fig. 4A,D–F). These results suggested that treatment with class I HDAC inhibitors + COX-1/COX-2 inhibitors + ICIs may activate CTL/NK cells to kill tumor cells in the TME and finally to reduce the tumor burden.

### Anticancer mechanism of a triple combination regimen containing chidamide + celecoxib (CC) + anti-PD-1 antibody

**Figure 5:**
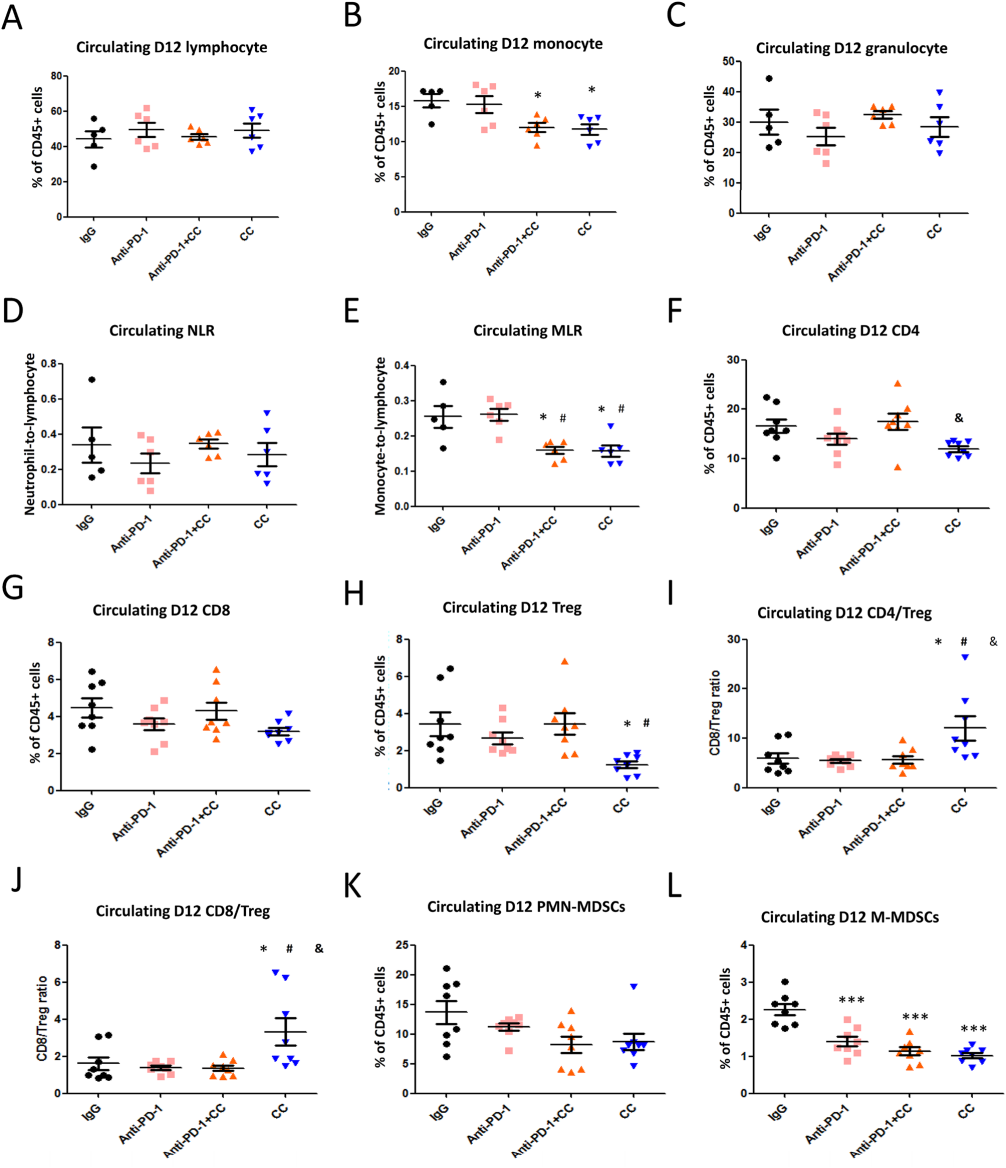
Populations of immune cells, including lymphocytes, Tregs and myeloid-derived MDSCs, in the blood circulation of CT26-bearing mice. CT26 tumor-bearing mice were orally administered a chidamide-k30 solution and celecoxib (50 mg/kg) once daily from days 10 to 22 (Day 10 mean tumor volume (TV), 220–240 mm^3^). The mice were treated with anti-PD-1 antibody (2.5 mg/kg, i.p.) once every 3 days on days 10, 13, 16, 19, and 22. Blood samples were obtained on day 12 after treatment for analysis of circulating cell populations. (**A–C**) Flow cytometric analysis of lymphocytes, monocytes, and granulocytes in peripheral blood. Results are shown as mean ± standard deviation (SD). *p < 0.05 or **p < 0.01 vs. anti-IgG. (**D,E**) Ratios of neutrophil-to-lymphocyte and monocyte-to-lymphocyte in peripheral blood by Flow cytometric analysis. Results are shown as mean ± SD. *p < 0.05 vs. anti-IgG; ^#^p < 0.05 vs. anti-PD-1. (**F–H**) Flow cytometric analysis of CD4^+^, CD8^+^, and Treg cell populations in peripheral blood. Results are shown as mean ± SD. ^&^p < 0.05 vs. anti-PD-1 + CD + C. (**I,J**) CD4^+^/Treg and CD8^+^/Treg ratios in peripheral blood by Flow cytometric analysis. Results are shown as mean ± SD. *p < 0.05 vs. anti-IgG, ^#^p < 0.05 vs. anti-PD-1, ^&^p < 0.05 vs. anti-PD-1 + CD + C. (**K,L**) Flow cytometric analysis of myeloid-derived PMN-MDSC and M-MDSC cell populations in peripheral blood. Results are shown as mean ± SD. ***p < 0.001 vs. anti-IgG (n = 6–8).

Next, we determined whether the combination of chidamide + celecoxib or triple combination of chidamide + celecoxib + anti-PD-1 antibody affected the T-cell population in peripheral blood and tumors. Blood samples were collected and isolated at day 12 after the above treatment and assessed by flow cytometry. Our findings showed no significant changes in circulating lymphocytes and granulocytes; however, monocytes were significantly reduced by approximately 22% after chidamide + celecoxib treatment and 25% after chidamide + celecoxib + anti-PD-1 antibody (Fig. 5A–C, SFig. 6A). However, anti-PD-1 antibody treatment did not significantly impact this cell population. Furthermore, neutrophil-to-lymphocyte (NLR) and monocyte-to-lymphocyte (MLR) ratios can effectively reflect the inflammation and immune status in vivo, which are reportedly associated with tumor progression and prognosis^29^. We analyzed the NLR and MLR ratios and revealed that the circulating MLR ratio decreased by approximately 26% after treatment with a combination of chidamide + celecoxib. After treatment with the triple combination of chidamide + celecoxib + anti-PD-1 antibody, the MLR ratio decreased by approximately 26% (Fig. 5D,E). Collectively, these results suggested that the chidamide + celecoxib regimen potently reduced monocytes in the blood circulation of the CT26-bearing mice model. Next, we determined whether the combination of chidamide + celecoxib affected the circulating T-cell population. Our results showed that CD4^+^ T cells and FoxP3^+^ Tregs were significantly reduced after treatment with the chidamide + celecoxib regimen and no significantly change in triple combination (Fig. 5F–H, SFig. 6B,C). It is well known that the CD4/Treg or CD8/Treg ratio promotes an immune response to tumors. Our findings revealed that the chidamide + celecoxib treatment regimen significantly increased the circulating CD4^+^/FoxP3^+^ Tregs and CD8^+^/FoxP3^+^ Tregs, suggested less immunosuppressive cells present in circulation (Fig. 5I,J). In addition, reportedly, myeloid-derived immature cells are often elevated in tumor-bearing hosts and have potent immunosuppressive activities. We observed that CT26 tumor-bearing mice presented reduced numbers of circulating M-MDSCs but not those of PMN-MDSCs in each treatment (Fig. 5K,L, SFig. 6D). M-MDSCs are positive correlation with tumor size (SFig. 6E). Based on these results, we concluded that the anti-PD-1 antibody effects were possibly attributed to the depletion of M-MDSCs. The effects demonstrated by the combination regimen of chidamide + celecoxib were possibly a result of M-MDSC and Treg depletion.

**Figure 6:**
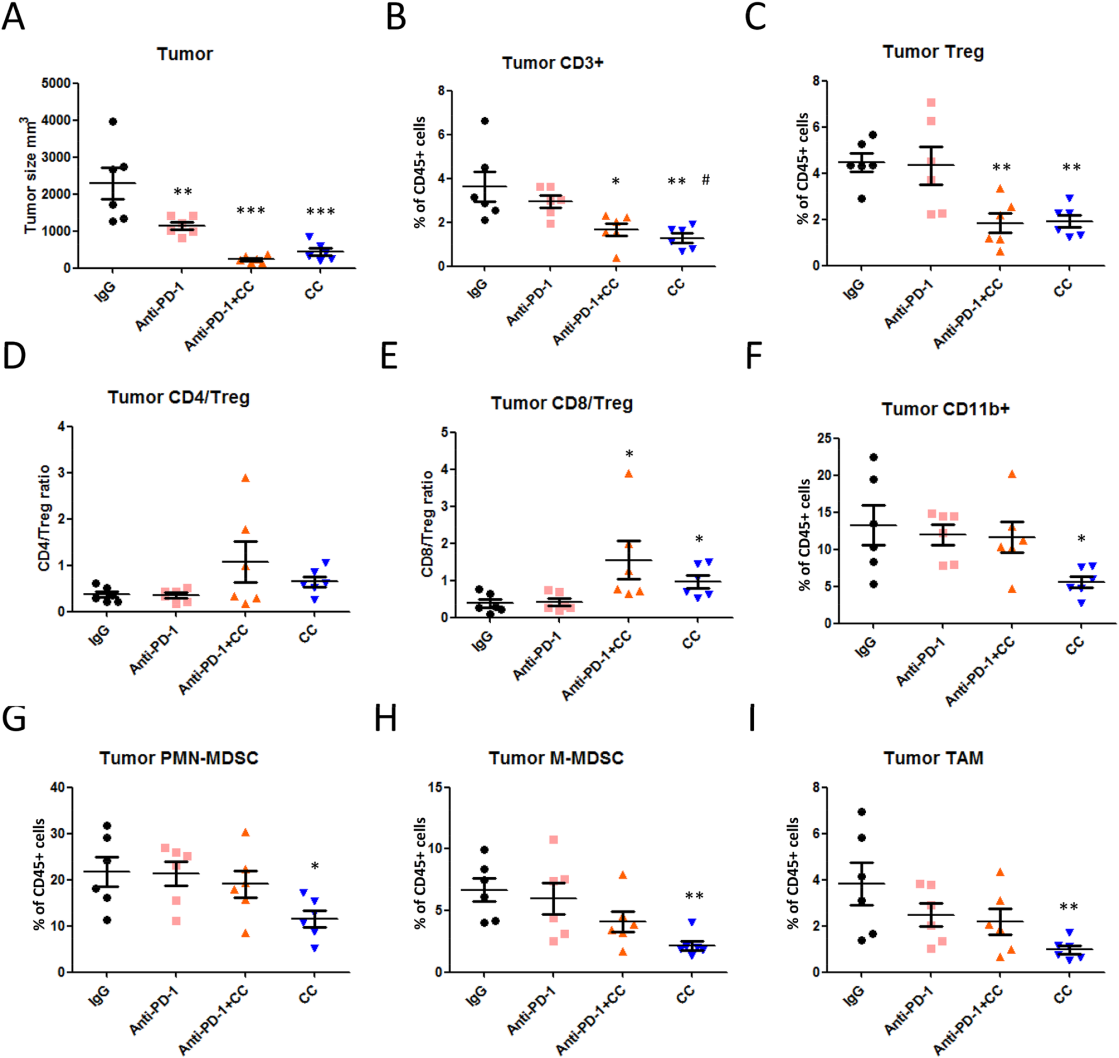
Infiltrating immune cell populations of lymphocytes and myeloid-derived MDSCs in tumors. (**A**) CT26 tumor-bearing mice were orally administered a chidamide-k30 solution and celecoxib (50 mg/kg) once daily from days 10 to 22 (Day 10 mean tumor volume (TV), 220–240 mm^3^). Endpoint tumor size is presented as TV (mm^3^) performed on day 12 after initial treatment. Results are shown as mean ± standard deviation (SD). **p < 0.01 or ***p < 0.001 vs. anti-IgG. Tumor-infiltrating lymphocytes were isolated by the Percoll gradient centrifugation method. (**B,C**) Percentages of CD3^+^ T cells and Tregs in tumors by flow cytometric analysis. Results are shown as mean ± SD. *p < 0.05 or **p < 0.01 vs. anti-IgG; ^#^p < 0.05 vs. anti-PD-1. (**D,E**) CD4/Treg and CD8/Treg ratios in tumors by flow cytometric analysis. Results are shown as mean ± SD. *p < 0.05 vs. anti-IgG. (**F**) Flow cytometric analysis of myeloid-derived CD11b^+^ cells in tumors. Results are shown as mean ± SD. *p < 0.05 vs. anti-IgG. (**G,H**) Flow cytometric analysis of myeloid-derived PMN-MDSC and M-MDSC cell populations in tumors. Results are shown as mean ± SD. *p < 0.05 or **p < 0.01 vs. anti-IgG. (**I**) Flow cytometric analysis of myeloid-derived Ly6C^+^/MHCll^+^ tumor-associated macrophages in tumors. Results are shown as mean ± SD. *p < 0.05 vs. anti-IgG.

Finally, to determine whether the combination of double or triple regiment impacts immune cell infiltration within the TME, we analyzed the defense presented by CD8^+^ and CD4^+^ T cells, immunosuppressive cells such as Tregs, and CD11b^+^ myeloid cells in the tumor-bearing mice. Tumors were isolated and quantified at day 12 after treatment (Fig. 6A). Tumor-infiltrating CD3^+^ T cells were reduced by approximately 56% and 67% after treatment with chidamide + celecoxib + anti-PD-1 antibody and chidamide + celecoxib, respectively (Fig. 6B; we also showed CD3^+^ T cells number in SFig. 7A). Tumor-infiltrating CD4^+^ T cells were reduced by approximately 62% and 55% after double or triple regiment treatment, respectively (SFig. 6F; we also showed CD4^+^ T cells number in SFig. 7B). Similarly, for tumor-infiltrating FoxP3^+^ Tregs, the cell number was decreased by approximately 60% and 57% after double or triple regiment treatment, respectively (Fig. 6C; we also showed FoxP3^+^ Treg cells number in SFig. 7D). Additionally, none of the treatment regimens altered cell numbers of tumor-infiltrating CD8^+^ T cells (SFig. 6G; we also showed CD8^+^ T cells number in SFig. 7C). However, the CD8/Treg ratio was significantly increased after double or triple regiment treatment (Fig. 6E; we also showed FoxP3^+^ Treg cells number in SFig. 7F). Next, we also investigated the CD8^+^ T cell populations in the tumor microenvironment on D12 in response to each treatment. The cell populations were determined as gated in SFig. 8. Tumors were isolated and quantified at day 12 after treatment (SFig. 9A). Combination treatment had no significant change in the infiltration of leukocytes and percentage of CD45^+^ cells, especially CD45^+^CD8^+^ T cells (SFig. 9B–D). Activated GzmB^+^CD8^+^ T cells, Ki67^+^CD8^+^ T cells, and INF-γ^+^CD8^+^ T cells were not significantly altered in each treatment group compared with IgG treatment (SFig. 9E–G). However, exhausted TIM-3^+^CD8^+^ T cells were significantly reduced by approximately 32.2% in the triple combination treatment group and PD-1^+^CD8^+^ T cell tend to be decreased after treatment compared with IgG control group (SFig. 9H–J). These results suggested that exhausted CD8^+^ T cells are downregulated by triple combination treatment in the CT26 model.

We next analyzed myeloid-derived cells and revealed that within tumors, CD11b^+^ cells were significantly reduced by 59% after chidamide + celecoxib treatment (Fig. 6F; we also showed CD11b^+^ cells number in SFig. 7G). Furthermore, we analyzed myeloid-derived MDSCs and showed PMN-MDSCs and M-MDSCs were reduced in tumors by 50% and 51%, respectively, after treatment with chidamide + celecoxib (Fig. 6G,H; we also showed PMN-MDSCs and M-MDSCs cells number in SFig. 7H,I). Finally, we analyzed the myeloid-derived TAMs, revealing that TAMs were reduced by 73% after treatment with chidamide + celecoxib (Fig. 6I; we also showed TAMs cells number in SFig. 7J). These data revealed that both chidamide + celecoxib and chidamide + celecoxib + anti-PD-1 antibody possibility activated immune cells by reducing immune suppression via Tregs, MDSCs, and exhausted T cells. A correlation assay revealed that the number of M-MDSCs positively correlated with the tumor size (SFig. 6E). These results demonstrated that chidamide + celecoxib therapy was a potent TME regulator.

### Effect of chidamide + celecoxib or triple combination of chidamide + celecoxib + anti-PD-1 antibody on gene expression in the tumor microenvironment

We aimed to determine the importance of combining regimen to induce an anti-tumorigenic response in the tumor microenvironment. To determine gene expression profiles of tumors collected from two treated mice of each treatment group, we analyzed whole-genome expression through a microarray platform containing coding and long intergenic non-coding RNAs. After filtering emission intensity data in the GeneSpring 12.6 software (Agilent Technologies), each case was classified according to its treatment, and all filtered genes were used for hierarchical clustering analysis based on genes with different gene expressions when compared with a control group treated with anti-IgG antibody.

Twofold higher and less than half of that of the anti-IgG Ab group was the inclusion criterial. Total of 589 genes up-regulated and 224 genes down-regulated were changed in the anti-PD-1 antibody, respectively. Moreover, total of 385 genes up-regulated and of 283 genes down-regulated were changed in the chidamide + celecoxib + anti-PD-1 antibody group, respectively (Table 1). Total of 861 genes up-regulated and of 389 genes down-regulated were changed in the chidamide + celecoxib group, respectively. Additionally, we performed GO analysis, demonstrating that genes associated with “response to interferon-gamma” in the biological process category were enriched (Table 2), demonstrating that enrichment of these gene sets is associated with TIL (tumor-infiltrating lymphocyte) activation in gene expression. Gene expression analysis of CT26 tumors revealed the induction of a plethora of immune-related pathways by chidamide + celecoxib (SFig. 10A–D). Treatment with chidamide + celecoxib resulted in the upregulation of proinflammatory IFN-γ response genes (SFig. 10A). Furthermore, chidamide + celecoxib increased the expression of granzyme genes positively associated with responses to CD8^+^ T and NK cells (SFig. 10B), as well as promoted M1 macrophage differentiation rather than M2 (SFig. 10C,D). Similar results were observed with the triple combination chidamide + celecoxib + anti-PD-1 treatment group. These results suggested that chidamide + celecoxib, with or without anti-PD1 antibody, induced “response to interferon-gamma”, resulting in unfavorable tumor growth in the tumor microenvironment.

**Table 1:** Gene chip analyses of gene expression in CT26 tumor grown in BALB/c mice revealed involvement of more than 1250 differentially expressed genes.

Comprising set	Upregulation	Downregulation
PD-1/IgG	589	224
PD-1 + CD + C/IgG	385	283
CD + C/IgG	861	389

**Table 2:** Top 5 significantly enriched GO terms.

	Description	P-adjust t	Number of genes
**PD-1/IgG**			
1	Cell chemotaxis	0.000562	18
2	Leukocyte migration involved in the inflammatory response	0.00297	5
3	Leukocyte chemotaxis	0.003186	14
4	Cellular response to interferon-gamm	0.01129	8
5	Response to interferon-gamma	0.01129	9
**PD-1 + CD + C/IgG**			
1	Response to interferon-gamma	5.73E−14	25
2	Cellular response to interferon-gamma	8.78E−14	22
3	Chemotaxis	3.58E−10	52
4	Regulation of inflammatory response	3.75E−10	37
5	Myeloid leukocyte migration	3.79E−09	26
**CD + C/IgG**			
1	Response to interferon-gamma	4.6E−13	20
2	Cellular response to interferon-gamma	4.6E−13	18
3	Regulation of inflammatory response	1.75E−10	29
4	Positive regulation of cytokine production	2.15E−10	33
5	Leukocyte migration	2.81E−10	28

The differentially expressed genes are summarized in the immune response. Gene Ontology enrichment analysis of biological processes for upregulated and downregulated genes between IgG vs anti-PD-1, vs anti-PD-1 + chidamide + celecoxib, and vs chidamide + celecoxib treated tumor samples were performed.

**Figure 7:**
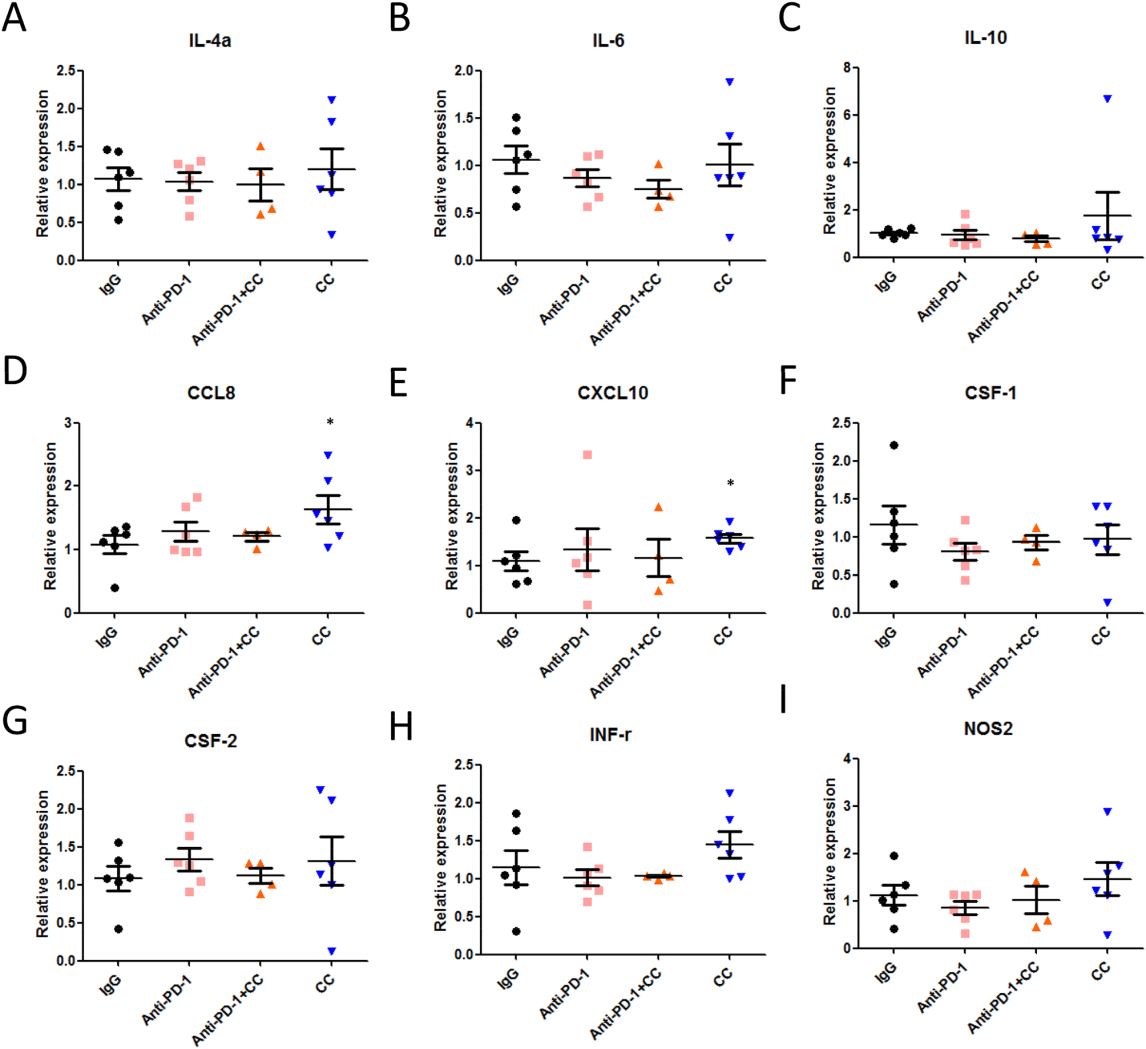
Determination of chemokines and NOS2 expression level in CT26 tumors. Total mRNA was extracted from anti-PD-1, anti-PD-1 + chidamide (CD) + celecoxib (C), CD + C, and IgG-treated tumors (n = 4–6 for each group). mRNA levels of genes were determined by qPCR. Data are presented as mean ± standard deviation (SD). *p < 0.05 by unpaired Student’s t-test. (**A**) IL-4a; (**B**) IL-6; (**C**) IL-10; (**D**) CCL8; (**E**) CXCL10; (**F**) CSF-1; (**G**) CSF-2; (**H**) INF-γ; (**I**) NOS2.

Furthermore, we determined whether chidamide + celecoxib suppressed tumor-induced myeloid-derived cell homing or immune cell infiltration to tumors. Primary tumors were collected and isolated on day 12 after chidamide + celecoxib treatment. We performed Q-PCR analyses to analyze the chemokine and MDSC marker expression in the CT26 tumor (Fig. 7A–I). The results revealed that chemokines, cytokines, and MDSC markers from CT26 tumors revealed significantly altered CCL8 and CXCL10 gene expression (Fig. 7D,E). Furthermore, to identify the role of chemokine and cytokine genes expression related to tumor growth, we analyzed the correlation between tumor size and gene expression level. The Q-PCR results from all the tumors of each treatment group showed that several chemokines and cytokines were positively correlated with tumor size, including IL-4a, IL-6, IL-10, CSF-1, CSF-2, INF*-*r, and NOS-2. Only CCL8 and CXCL10 showed a negative correlation with tumor size (SFig. 11A–I). These results suggested that chidamide + celecoxib presented antitumor activity by increasing CCL8 and CXCL10 gene expression (SFig. 11D,E) subsequently probably recruiting CTL cells in the TME. Chidamide + celecoxib combined with anti-PD-1 antibody demonstrated antitumor activity by downregulating multiple gene expression levels, suppressing immune cells in the tumor microenvironment.

## Discussion

In this study, by using a combination treatment strategy it enabled us to understand the effect of chidamide plus celecoxib as a combination regimen (CC-01), or further with an anti-PD-1 antibody as a triple combination regimen, on the immune cells in the TME of CT26-bearing mice, which resulted in enhanced antitumor responses. CC-01 or the triple combination regimen (CC-01 + anti-PD-1 antibody) demonstrated potent eradication of primary tumors by mediating the suppression of Tregs, myeloid-derived cells, and TAMs (Figs. 2, 3, 4), decrease of exhausted CD8^+^ T cells (SFig. 8), and increased ratios of CD4^+^ T/Treg and CD8^+^T/Treg (Figs. 5, 6). Additionally, we demonstrated that the formulation of a triple combination of class I HDAC inhibitors + COX-1/COX-2 inhibitors + ICIs would provide a higher anti-tumorigenic response (Fig. 4). Finally, the induction of immune activation by chip assay revealed that CC-01 or the triple combination regimen activated IFN-γ responsive genes, M1 macrophage and CD8 related genes (SFig. 10, Tables 1, 2). We also identified CC-01-induced CXCL10, whose gene expression level was negatively correlated with tumor size (Fig. 6, SFig. 11).

Immune checkpoint inhibitors, such as Anti-PD-1 or anti-PD-L1 mAbs, have improved overall survival (OS) of patients with various types of cancers^30^. However, not a few patients fail to achieve clinical benefit. Myeloid-derived suppressor cells (MDSCs) is a heterogeneous immature population of myeloid cells partly influencing the efficacy of immunotherapies. These cells not only directly suppress T cell but mediate a potently immunosuppressive network within tumor microenvironment to attenuate the anti-tumor response. According to present studies, PD-1 antibody performed moderate anti-tumor effect possibly by regulating lymphocyte activation and attenuating circulating M-MDSC. The results of previous study on clinical responders to ipilimumab therapy showed a significantly lower percentage of M-MDSC in the peripheral blood as compared to non-responders^31^. In addition, macromolecular therapeutic antibodies are well known to display slow extravasation and incomplete penetration into tumors, potentially protecting cancer cells from therapeutic effects^32^. However, our studies above collectively demonstrated that the poor anti-cancer effect of anit-PD-1antibody may be not only due to its large size but also the lack of its effect on MDSC and other immunosuppressive cells in tumor.

Celecoxib is an anti-inflammatory drug by inhibiting COX2, with potent antitumor activity in the treatment and prevention of cancer^33^. PGE2 can activate several key immune-suppressive cells present in the TME such as Treg, M-MDSC, and TAM. However, monotherapy fail to achieve preclinical benefit^34^. Currently our study also demonstrated inhibition of COX has no synergy with anti-PD-1 blockade in inducing eradication of tumors (Fig. 1C). In addition, chidamide monotherapy performed poor anti-cancer effect on CT26 tumor-bearing mice, but class I HDAC inhibitor have potential to attenuate immunosuppressive cells in TME^28^. Therefore, we were interested in evaluating a combination of celecoxib + chidamide. In our current study, we revealed the optimal dosage of chidamide (50 mg/kg) and celecoxib (50 mg/kg) in the combination regimen. We observed that CC-01 demonstrated a potent immune response by down-regulating the cell number of M-MDSCs, Tregs, and TAMs in TME. Furthermore, we demonstrated that CC-01 in combination with immune checkpoint blockade, as a triple regimen, presented a significantly increased immune response in CT26 tumors.

To confirm the anticancer mechanisms of the triple combination regimen through immunomodulation, we evaluated several class I HDAC inhibitors (chidamide, entinostat, and mocetinostat), COX-1/COX-2 inhibitors (aspirin, ibuprofen, and celecoxib), and ICIs (anti-PD-1/anti-CTLA-4 antibody) in different regimens. In the triple regimens investigated, anti-CTLA-4 antibody demonstrated great efficacy probably by suppressing the inhibition of dendritic cell activity via CTLA-4 blocking on Tregs, and subsequently activating T cells in lymph nodes/tissues^35^, when synergistically combined with CC-01 treatment. Furthermore, the anti-PD-1 antibody plus CC-01 triple combination regimen demonstrated similar results, probably by direct PD-1 blockade to augment T cell activation synergistically with CC-01^35^. Both anti-CTLA-4 antibody and anti-PD-1 tested were highly effective in the triple combination. Finally, it has been known that several class I HDAC inhibitors (valproic acid, panobinostat and entinostat) modulate PD-L1 expression^36^, suggested possible benefit when combined with immune checkpoint blockade can be highly effective to achieve preclinical benefit in current study. However, PD-1^+^ regulatory T cells amplified by PD-1 blockade promote hyperprogression of cancer was demonstrated in animal study in which murine Tregs that were deficient in PD-1 signaling were more proliferative and immunosuppressive^37^. Therefore, in our study the circulating Tregs shown in flow cytometry data was significantly different in cell number between CC-01 and triple combination (Fig. 5H), suggesting that anti-PD-1 neutralized CC-01 attenuation on Treg cell number. Our findings indicated that these class I HDAC inhibitors possessed a similar mechanism to antitumor activation. The efficacy of the ibuprofen-containing triple combination regimen was not significant owing to its lower half-life than COX-2 inhibitor celecoxib, partially reducing the tumor volume and failing to effectively eradicate primary tumors. Similar results were observed with aspirin-containing triple combination treatment, with failure to demonstrate remarkable primary tumor eradication. In summary, because tumors are highly heterogeneous and dynamic in their own environment, differences in tumor volume may respond differently due to different cell composition. The previous study demonstrated that lymphocyte and CD8^+^ T cells number decreased in ≧ 8 cm undifferentiated pleomorphic sarcomas (UPS) than < 8 cm UPS^38^. We have also observed that when the smaller the tumors at the starting point of administration, the better the efficacy would show, which may be due to the less levels of circulating immunosuppressive cells homing to tumor microenvironment in small size tumor^39^. In order to correct this issue, the statistics of Figs. 2, 3 and 4 were collected and the results showed that the response rate (CR + PR) of anti-PD-1was about 25% (7/27), CC-01 was about 74% (20/27), and the triple combination was about 93% (27/30). The anti-tumor response of chidamide or celecoxib alone is very weak, and the combination of the two or further combined with ICIs can significantly increase the anti-tumor activity, showing a strong synergistic effect. The addition of anti-PD-1 to CC-01 combination can increase the tumor suppressing ability by about 20% when compared to CC-01 combination, however strongly enhance the anti-tumor response by about 70% when compared to anti-PD-1 alone. Our findings suggested that the addition of CC-01 may be the optimal composition of immunotherapeutic combinations in our study to achieve maximal sensitization of immune activation or enhance ICI immunotherapy.

The anti-tumorigenic effect of CC-01 or CC-01 + anti-PD-1 antibody triple combination by activating immune cells in mice were mainly observed in wild mice but not in nude mice and CD8^+^ T cell-deleted wild mice administered by anti-CD8 antibody, suggesting that anti-tumorigenic actions of combination regimens required CD8^+^ T cell activation in the TME to kill tumors. We also found that anti-cancer activity after treatment with anti-PD-1 antibody, CC-01 or CC-01 + anti-PD-1 antibody was boosted by CD4^+^ T cell deletion, suggesting that CD4^+^Treg decrease was important factor in immunotherapeutic effect. In addition, in the in vitro study, CT26 cells treated with chidamide demonstrated suppressed tumor proliferation, necessitating approximately 10 μM. This 10 μM concentration was not achieved in the peripheral blood of CT26-bearing BALB/C wild type and nude mice following the oral administration of 50 mg/kg chidamide (PK data not shown). The CC-01 and CC-01 + anti-PD-1 significantly reduced FoxP3^+^Tregs through both mobilization and homing in CT26-bearing mice, resulting in an increased CD8^+^/Treg ratio and immune activation in tumors. This finding was consistent with entinostat, which enhances the acetylation of STAT3, impairs FoxP3 expression and Treg function, and impairs the suppressive capacity of Tregs^40^. In addition, tumor-infiltrating CD8^+^IFN-γ^+^, CD8^+^Ki67^+^ and CD8^+^GzmB^+^ T cells were not significantly altered by each treatment in tumor. CD8^+^TIM-3^+^ T cell were significantly suppressed by the CC-01 + anti-PD-1 combination regimen treatment and CD8^+^PD-1^+^ T cells were shown to have a decreased trend after treatment (SFig. 9). Furthermore, CC-01 significantly reduced both PMN- and M-MDSC mobilization and homing to the TME. This finding was consistent with combined treatment with entinostat and anti-PD-1 antibody that reduces the tumor burden in murine models of melanoma and lung cancer, and in some cases by reducing the number of MDSCs^28,41,42^. Therefore, we postulate that CC-01 activated antitumor immunity by reducing tumor-infiltrating suppressor cells as a “TME regulator”, indirectly activating CTL by eliminating tumor immune evasion. In the chip data, IFN-γ, TNF-α, GzmB were predominately activated by the CC-01 or triple combination regimen treatment. Conversely, IFN-γ has also been shown to suppress genes related to M2-like functions in macrophages^43^. In addition, we also found chidamide activated major histocompatibility complex I (MHC-I) and IFN-γ expression in tumor (data not shown). In addition, we further demonstrated that CC-01 induced CXCL10 gene expression in tumor, suggesting this treatment regimen may increasing unknown population in tumor-infiltrating lymphocyte (TIL), possibly natural killer (NK)-cells^44^. Collectively, these results demonstrated that the CC-01 or CC-01 + anti-PD-1 combination regimen primarily reduced the tumor burden by directly improving the TME components and preventing T cells exhaustion after IFN-γ activation.

This study presented has some limitations. First, evidence regarding the mechanism of CC-01 reduced mobilization and phenotyping of immune-suppressive cells (Tregs, MDSCs) are lacking. Mobilization of inflammatory monocytes from the bone marrow into blood circulation is mediated via chemokines and their receptor^45^. Therefore, we speculate that CC-01 may reduce chemokine production in tumors, resulting in reduced Treg and MDSC mobilization. A clinical study investigating chidamide administration 30 mg BIW has revealed that adverse events observed in ≥ 10% of patients were thrombocytopenia (51%), leukopenia (40%), and neutropenia (22%) in phase II clinical trial in patients with relapsed or refractory peripheral T-cell lymphoma^46^. Therefore, we postulate that chidamide predominantly reduced white blood cell mobilization. Based on the dose conversion between animal and human, dosage of chidamide 50 mg/kg/day in mice is to be about 4.06 mg/kg/day in humans (the exchange factor is 12.3^47^). However according to clinical dosage 0.14 mg/kg/day (30 mg/60 kg, BIW) in human, the dose 4.06 mg/kg/day is 29 times the dose used in humans. Also the repeated dose PK analysis conducted in clinical trial revealed that BIW dosing schedule increased drug exposure^48^. Therefore, the clinical dosage of chidamide may achieve the anti-cancer efficacy of combination treatment for cancer patients. Second, we must consider that CC-01 regimen has a direct effect on antitumor immune cells. Chidamide has both epigenetic modulator and immunomodulatory properties. Gene expressions can be turned on or off under different pathological and physiological conditions. As an epigenetic modulator, chidamide may have specific targets to attenuate pathology-induced gene expression. Additionally, chidamide was shown to enhance the activities of NK cells and antigen-specific CD8^+^ cytotoxic T-lymphocyte (CTL) mediated cellular antitumor immunity^18–22^. Therefore, we need to consider the possibility that CC-01 has a direct effect on antitumor immune cell activation or immune-suppressive cell inhibition in TME. Third, based on the limited data, CC-01 did not significantly altered the infiltration of CD4^+^ or CD8^+^ T cells in tumor but showed trend of decrease in exhausted T cells. Also, it is not known which type of CTLs is affected by CCL8 and CXCL10 gene expression, possibly NK and CD8^+^ population. We need to address this issue in the furfure.

## Conclusion

In summary, we revealed a critical role of the CC-01, demonstrating significant activation of antitumor immunity by attenuating circulating Tregs, myeloid-derived cells, tumoral myeloid-derived cells, exhausted T cells, as well as TAMs. The development of the CC-01 as a pharmacological strategy for enhancing the immune response for superior anticancer efficacy provides a potentially persistent therapeutic response in several advanced cancer patients. Our study defines the cellular targets of CC-01 using a comprehensive panel of immune cells and confirms the requirement of IFN-γ signaling in the immune response. Furthermore, we showed the synergistic effect of the CC-01 in combination with anti-PD-1 antibody induced immunogenic response in CT-26-bearing mice. We revealed that a CC-01 can increase the activation of immune subsets, using doses of epigenetic therapy that are clinically relevant and can be immediately applied in clinical trials. In a currently ongoing phase Ib trial, the CC-01 is under investigation in patients with metastatic colorectal cancer who had progression or were intolerant of at least two lines of systemic therapies.

## Supplementary Information

Supplementary Figures.

## Data Availability

The datasets used and/or analyzed during the current study are available from the corresponding author on reasonable request.

## Abbreviations

COX2: Cyclooxygenase 2
CTL: Cytotoxic T-lymphocyte
DC: Dendritic cells
EM: Effector memory
Gzm: Granzyme
HDAC: Histone deacetylase
HDACi: HDAC inhibitor
IFN-γ: Interferon-gamma
i.p.: Intraperitoneally
MDSC: Myeloid-derived suppressor cells
MHC-I/MHCII: Major histocompatibility class I/class II
MS-275: Entinostat
NK: Natural killer
PBMC: Peripheral blood mononuclear cells
PD-1: Programmed cell death protein-1
PD-L1: Programmed cell death ligand-1
PGE2: Prostaglandin E2
PTCL: Peripheral T-cell lymphoma
RE: Response
RT: Room temperature
s.c.: Subcutaneously
SD: Standard deviation
TAMs: Tumor-associated macrophages
TIL: Tumor-infiltrating lymphocyte
TME: Tumor microenvironment
Treg: Regulatory T cell
WBCs: White blood cells
IL-4a: Interleukin 4 receptor alpha
IL-6: Interleukin 6
IL-10: Interleukin 10
CCL8: Chemokine ligand 8
CXCL10: C-X-C motif chemokine ligand 10
CSF-1: Colony-stimulating factor-1
INF-γ: Interferon-γ
NOS2: Nitric oxide synthase 2
