# Supplementary material for: CC-01 (chidamide plus celecoxib) modifies the tumor immune microenvironment and reduces tumor progression combined with immune checkpoint inhibitor
Source: Sci Rep. 2022 Jan 20;12:1100. doi: 10.1038/s41598-022-05055-8 (PMC8776878; doi:10.1038/s41598-022-05055-8)
Supplement: Supplementary file 1 — Supplementary Figures. [file 41598_2022_5055_MOESM1_ESM.docx]

Research Article

CC-01 (chidamide plus celecoxib) modifies the tumor immune microenvironment and reduces tumor progression combined with immune checkpoint inhibitor

Jia-Shiong Chen^1^, Cheng-Han Chou^2^, Yi-Hong Wu^2^, Mu-Hsuan Yang^3^, Sz-Hao Chu^3^, Ye-Su Chao^1^, Chia-Nan Chen^1*^

**Author affiliations**

^1^ New Drug Research and Development Center, GNT Biotech & Medicals Corporation (GNTbm), Taipei, Taiwan

^2^ Department of Biology, GNT Biotech & Medicals Corporation (GNTbm), Taipei, Taiwan

^3^ Department of Chemistry, GNT Biotech & Medicals Corporation (GNTbm), Taipei, Taiwan

*** Correspondence: Chia-Nan Chen: alex.chen@gntbm.com.tw**

**Supplemental data**

**
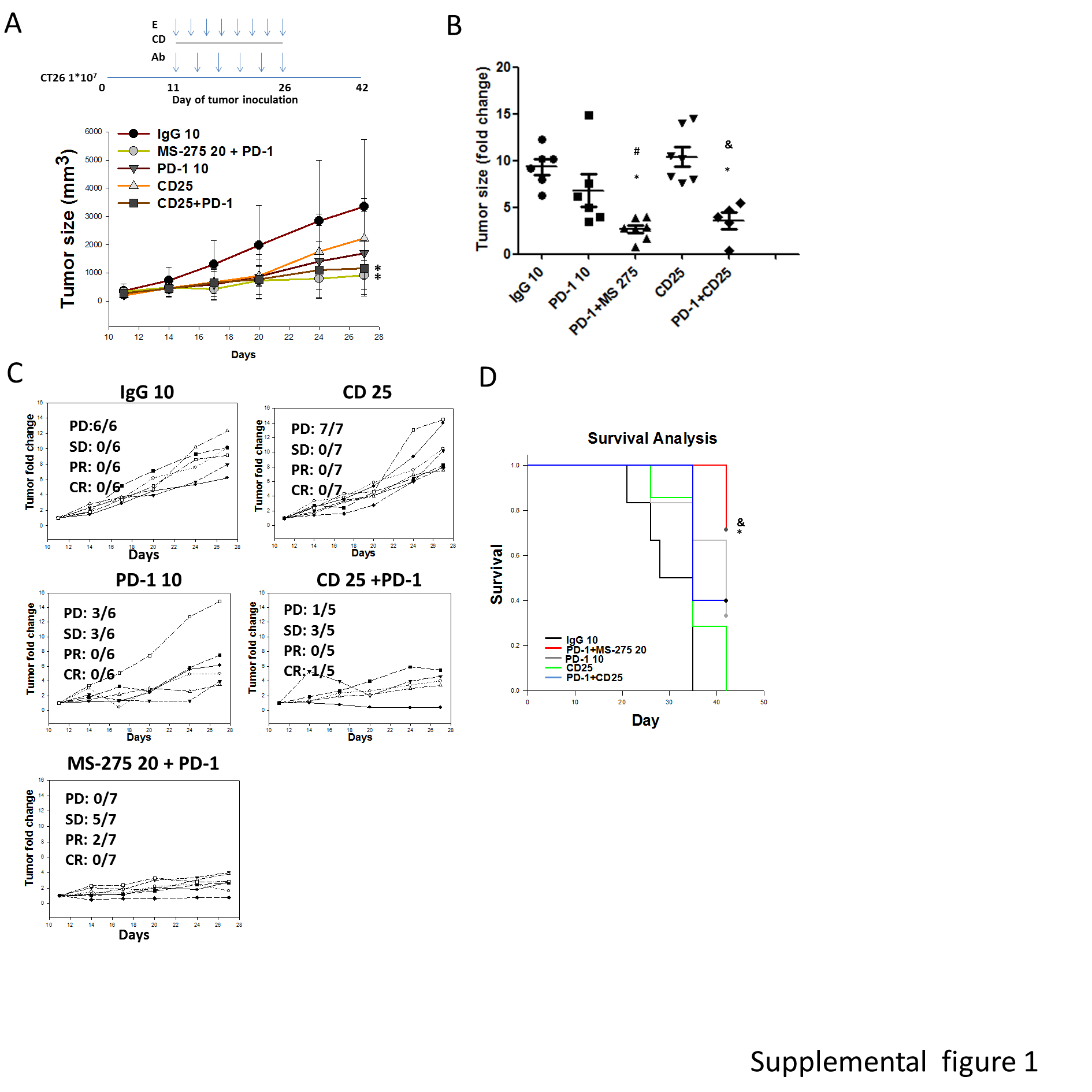
**

**S-Figure 1. Chidamide improves anti–PD-1-induced immunotherapy in an** **allograft CT26 mouse model**

**A**. CT26 tumor-bearing mice were orally administered a chidamide-k30 solution once daily (CD, 25 mg/kg) from days 11 to 26 or entinostat (E,20 mg/kg/2 days) once every 2 days from days 11 to 26 (Day 11 mean tumor volume (TV), 200-250 mm^3^). Arrows indicate the time points at which mice were treated with the anti–PD-1 antibody or IgG as a negative control. The mice were treated with anti–PD-1 antibody (10 mg/kg, i.p.) once every 3 days on days 11, 14, 17, 20, 23, and 26. Entinostat (MS-275), as a positive control, was administered in combination with the anti–PD-1 antibody following the indicated schedules. **B.** Endpoint tumor size in fold change. Results are shown as mean ± stand error of the mean (SEM). *p<0.05 vs. anti-IgG; #p<0.05 vs. anti–PD-1; &p<0.05 vs. CD25. **C.** Individual tumor growth graphs for CT26 tumor-bearing mice (n=5-7). Number of tumor-free mice per total number of mice is shown at the top left corner of each panel. **D.** Survival study of all the treatment groups.

**
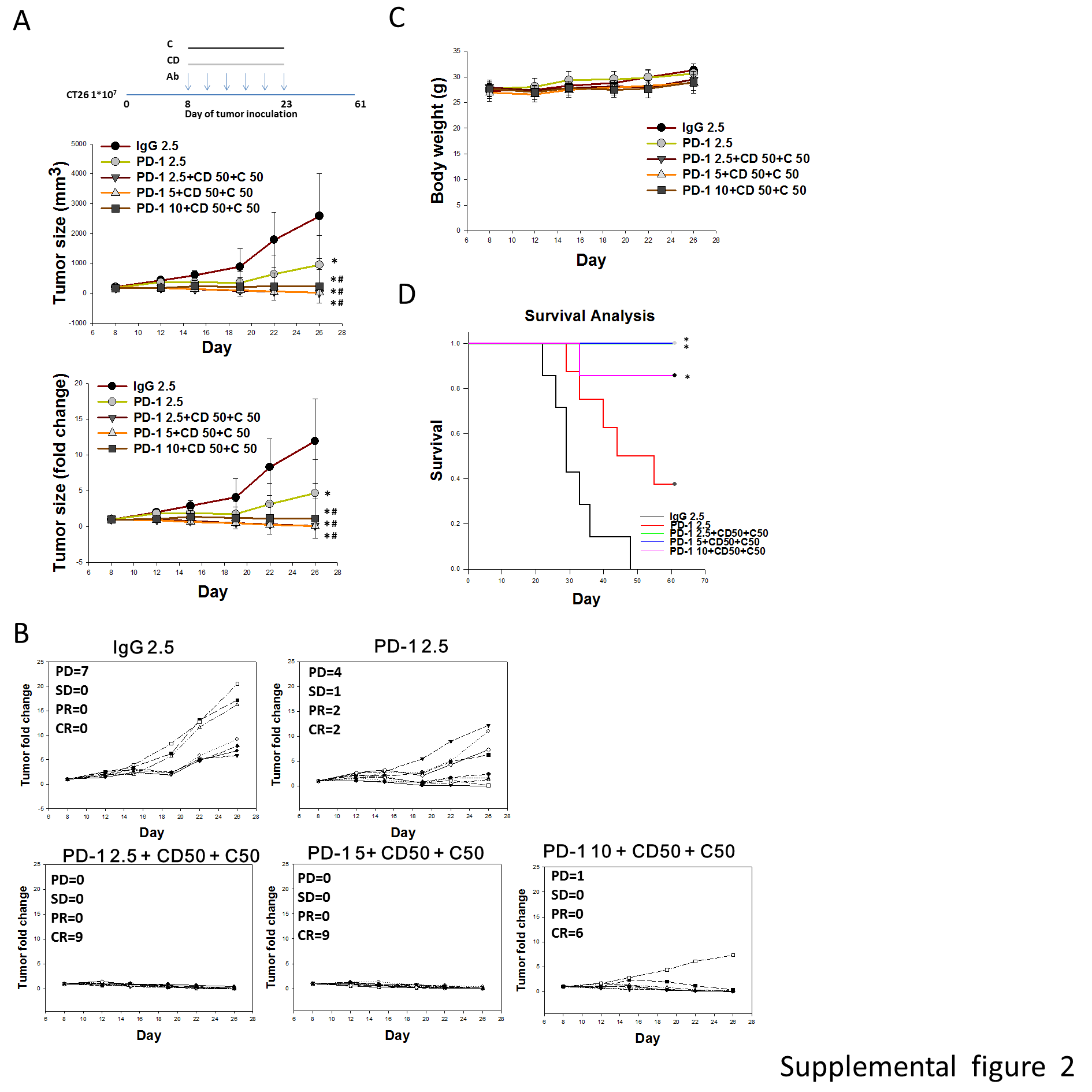
**

**S-Figure 2. Chidamide + celecoxib combined with an increasing dose of anti–PD-1-induced immunotherapy in an allograft CT26 mouse model**

**A.** CT26 tumor-bearing mice were orally administered a chidamide-k30 (CD; 50 mg/kg) once daily from days 8 (mean tumor volume (TV), 200-250 mm^3^) to 23, and celecoxib (C; 50 mg/kg) once daily from days 8 to 23. Arrows indicate the time points at which mice were treated with increasing doses of anti–PD-1 (2.5, 5, 10 mg/kg). The mice were treated with anti–PD-1 antibody (i.p.) once every 3 days on days 8, 11, 14, 17, 20, and 23. Endpoint tumor size in fold change. Results are shown as mean + standard deviation (SD). *p<0.05 vs. anti-IgG; #p<0.05 vs. anti–PD-1. **B.** Individual tumor growth graphs for CT26 tumor-bearing mice (n=7-9). Number of tumor-free mice per total number of mice is shown at the top left corner of each panel. **C.** Body weight of mice in each treatment group was recorded. **D.** Survival study of all the treatment groups.

**
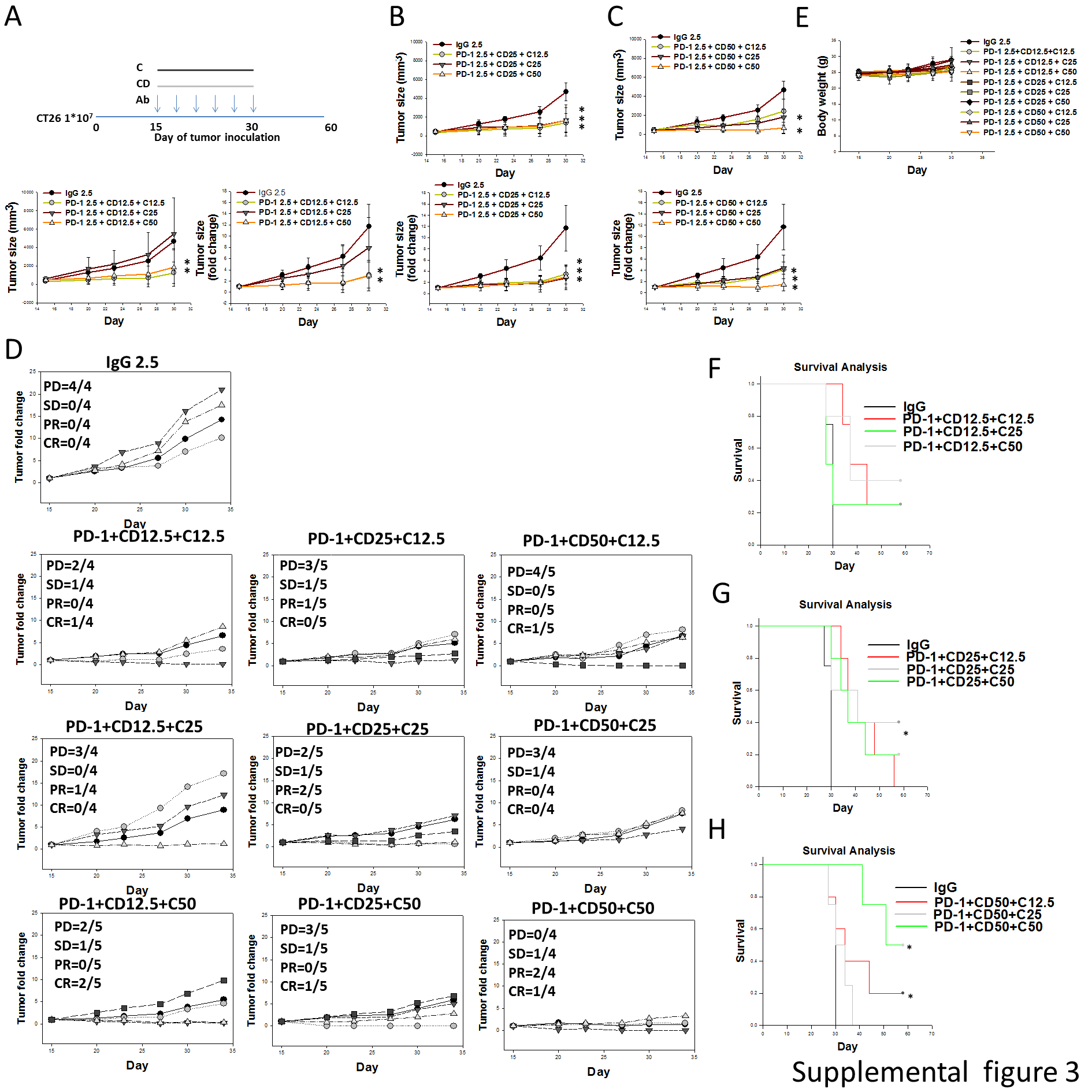
**

**S-Figure 3. Optimal doses of chidamide + celecoxib enhance anti–PD-1-induced immunotherapy in an allograft CT26 mouse model**

**A to C**. CT26 tumor-bearing mice were orally administered a chidamide-k30 solution (CD; 12.5, 25, 50 mg/kg) once daily from days 15 (mean tumor volume (TV), 250-300 mm^3^) to 30, and celecoxib (C; 12.5, 25, 50 mg/kg) once daily from days 15 to 30. Arrows indicate the time points at which mice were treated with anti–PD-1. The mice were treated with anti–PD-1 antibody (2.5 mg/kg, i.p.) once every 3 days on days 15, 18, 21, 24, 27, and 31. Endpoint tumor size in fold change. Results are shown as mean ± standard deviation (SD). *p<0.05 vs. anti-IgG; #p<0.05 vs. anti–PD-1; &p<0.05 vs. PD-1+CD50. **D.** Individual tumor growth graphs for CT26 tumor-bearing mice (n=4-5). Number of tumor-free mice per total number of mice is shown at the top left corner of each panel. **E.** Body weight of mice in each treatment group was recorded. **F-H.** Survival study after treatments with increasing doses of chidamide + celecoxib, combined with anti–PD-1 antibody.


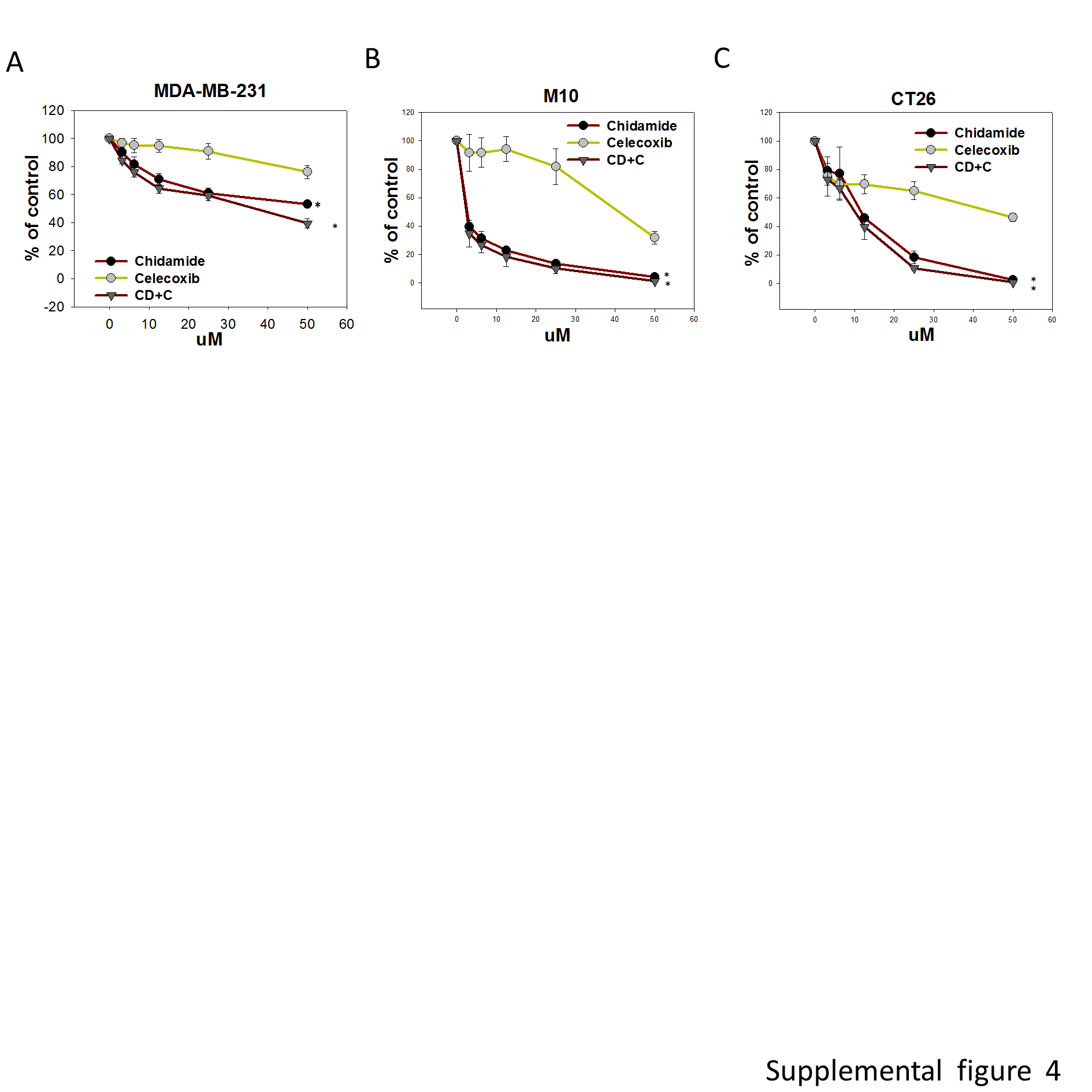


**S-Figure 4.** **Growth inhibitory effects of chidamide combined with celecoxib on MDA-MB-231, M10, and CT26 cells**

**A.** Growth curves of breast cancer cells MDA-MB-231 following treatments with chidamide, celecoxib, or chidamide + celecoxib for 72 h. **B.** IC_50_ values of normal cells M10 treated with chidamide, celecoxib, or chidamide + celecoxib for 72 h. Cell proliferation was determined with the MTT assay. **C.** IC_50_ values of mice colon cancer CT26 cells treated with chidamide, celecoxib, or chidamide + celecoxib for 72 h. Cell proliferation was determined using the MTT assay. The results were derived from three independent experiments performed in triplicate.


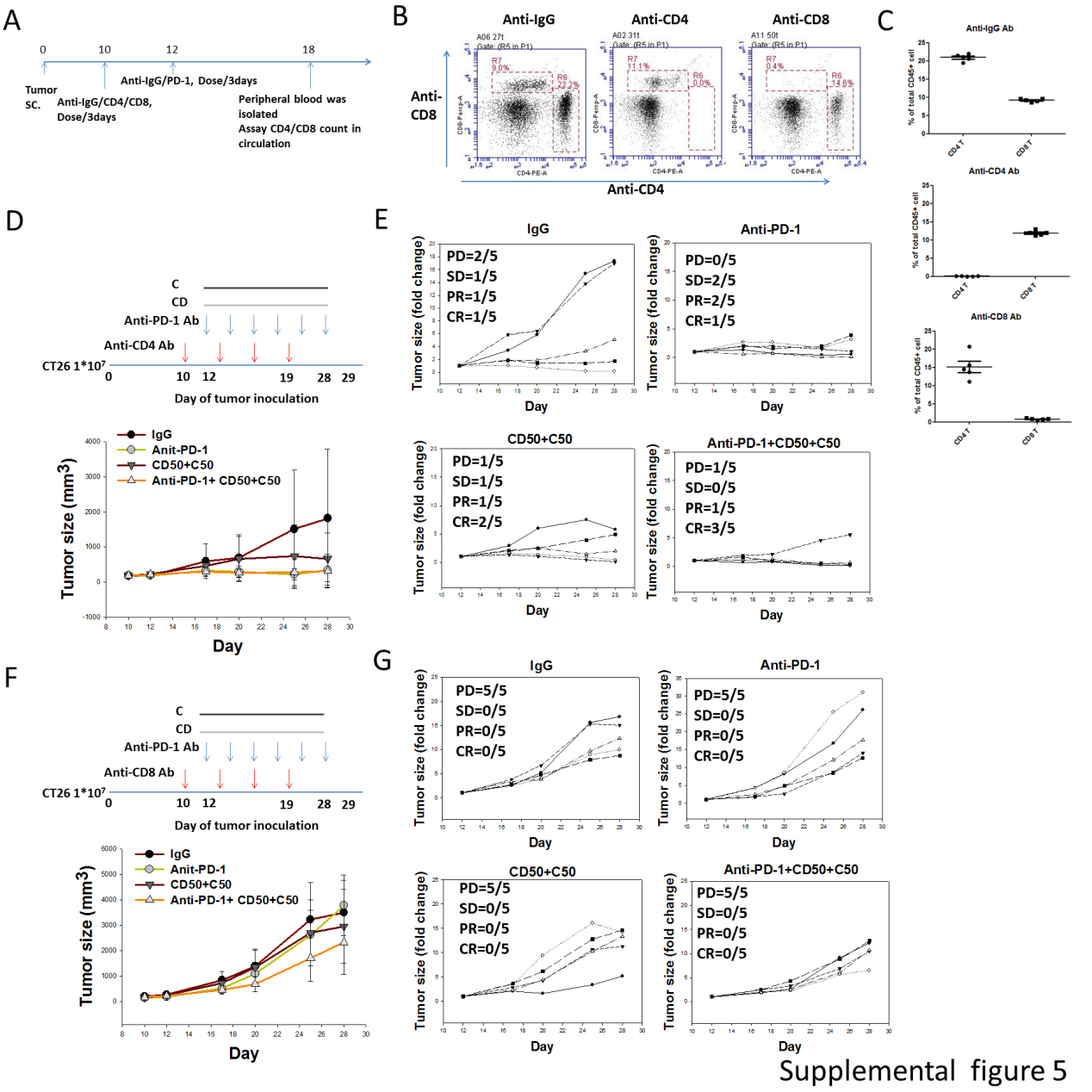


**S-Figure 5. Antitumor activity of chidamide + celecoxib in combination with anti-PD-1 antibody was tested under CD4^+^ or CD8^+^ T cell-depleted condition in the CT26 model.**

**A.** Mice, inoculated with CT26 cells, were administered with anti-CD4 or anti-CD8 antibody or control IgG on Day 10 and thereafter every 3 days for a total of 4 times. **B.** **and C.** For flow cytometric analysis, the percentage of CD4^+^ and CD8^+^ T cells in the blood was determined and quantified on day 9 after treatment, as shown in A.

**D.** Two days after the first injection of anti-CD4, mice were randomized into 4 groups with 5 mice in each group and an average tumor size 209 mm^3^, and each group was treated with vehicle, anti-PD-1, CD50+C50 or anti-PD-1+ CD50+C50, respectively. **E.** Individual tumor growth in each treatment group. The tumor assessment of each treatment group is shown at the top left corner of each panel.

**F.** Two days after the first injection of anti-CD8 antibody, mice were randomized into 4 groups with 5 mice in each group and an average tumor size 209 mm^3^, and each group was treated with vehicle, anti-PD-1, CD50+C50 or anti-PD-1+ CD50+C50, respectively. G. Individual tumor growth in each treatment group. The tumor assessment of each treatment group is shown at the top left corner of each panel.

Results are shown as mean +SD. *, P<0.05, Dunnett’s test vs. control IgG (n = 5).


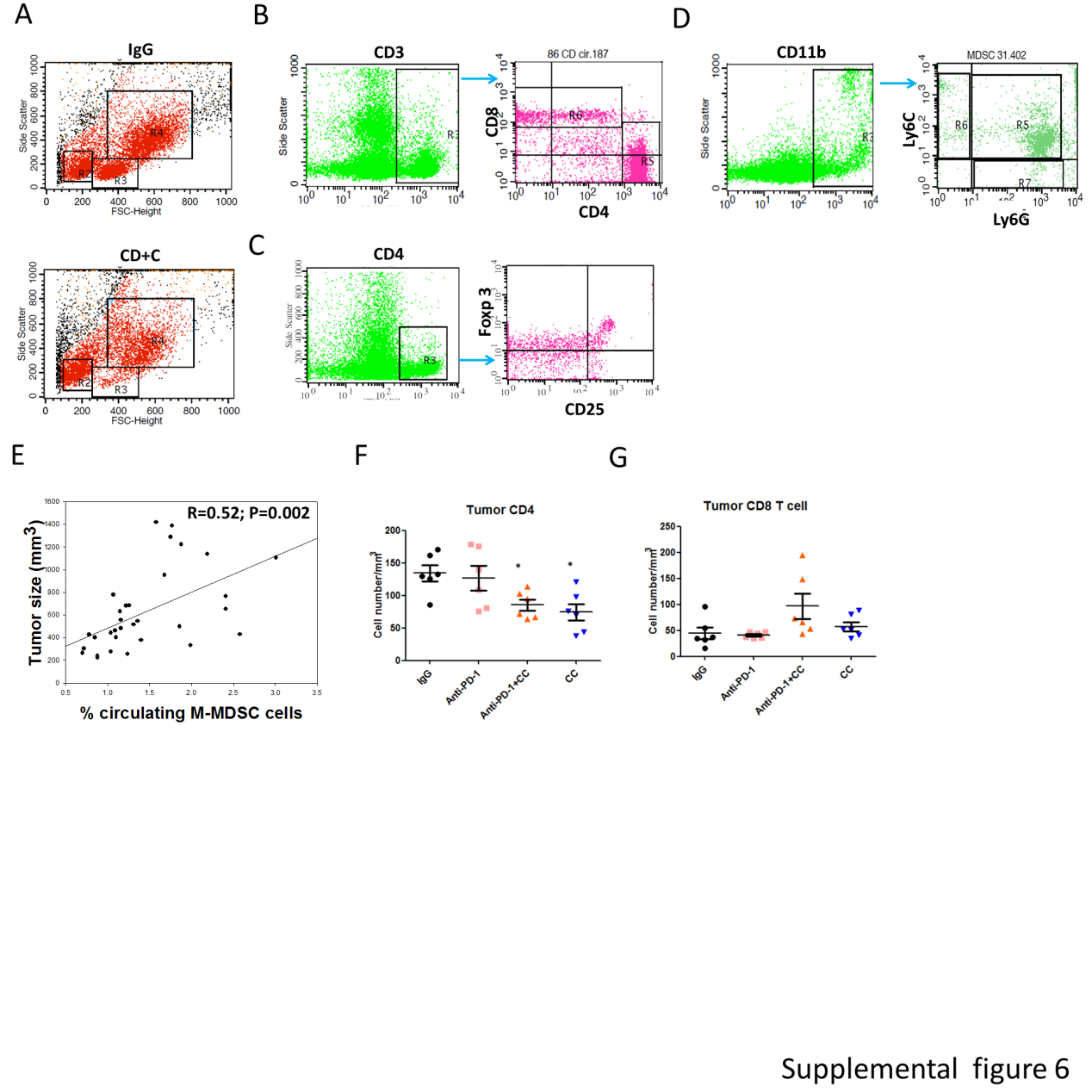


**S-Figure 6. Flow cytometric analysis of treatment-induced infiltration of immune cell subsets in BALB/c mice bearing CT26 tumors**

**A.** Flow cytometric gating strategy for lymphocyte, monocyte, and granulocyte subsets in mice peripheral blood. **B.** Flow cytometry analysis of CD3 T cell were stained for the T cell marker (CD45^+^/CD3^+^) , CD4 T cell (CD45^+^/CD3^+^/CD4^+^), and CD8 T cell (CD45^+^/CD3^+^/CD8^+^). **C.** Flow cytometry analysis of Treg cell (CD45^+^/CD4^+^/CD25^+^/Foxp^+^). **D.** Flow cytometry analysis of granulocyte-derived PMN-MDSC (CD45^+^/CD11b^+^/LY6G^+^) and monocyte-derived M-MDSS (CD45^+^/Cd11b^+^/LY6C^+^/LY6G^-^). Representative dot plot is shown. Gates were set based on isotype controls. Numbers represent the percentages from the parental populations gated. The gating strategy used to analyze the samples is illustrated. **E and F.** The status of infiltrating CD4^+^ and CD8^+^ cells in CT26 tumors isolated from BALB/c mice after treatment. **G.** Analyses were performed by analyzing antitumor activity data and flow cytometry data. The correlation between the CT26 tumor size and circulating M-MDSCs from all the treatment groups was analyzed by SigmaPlot Regression Wizard.


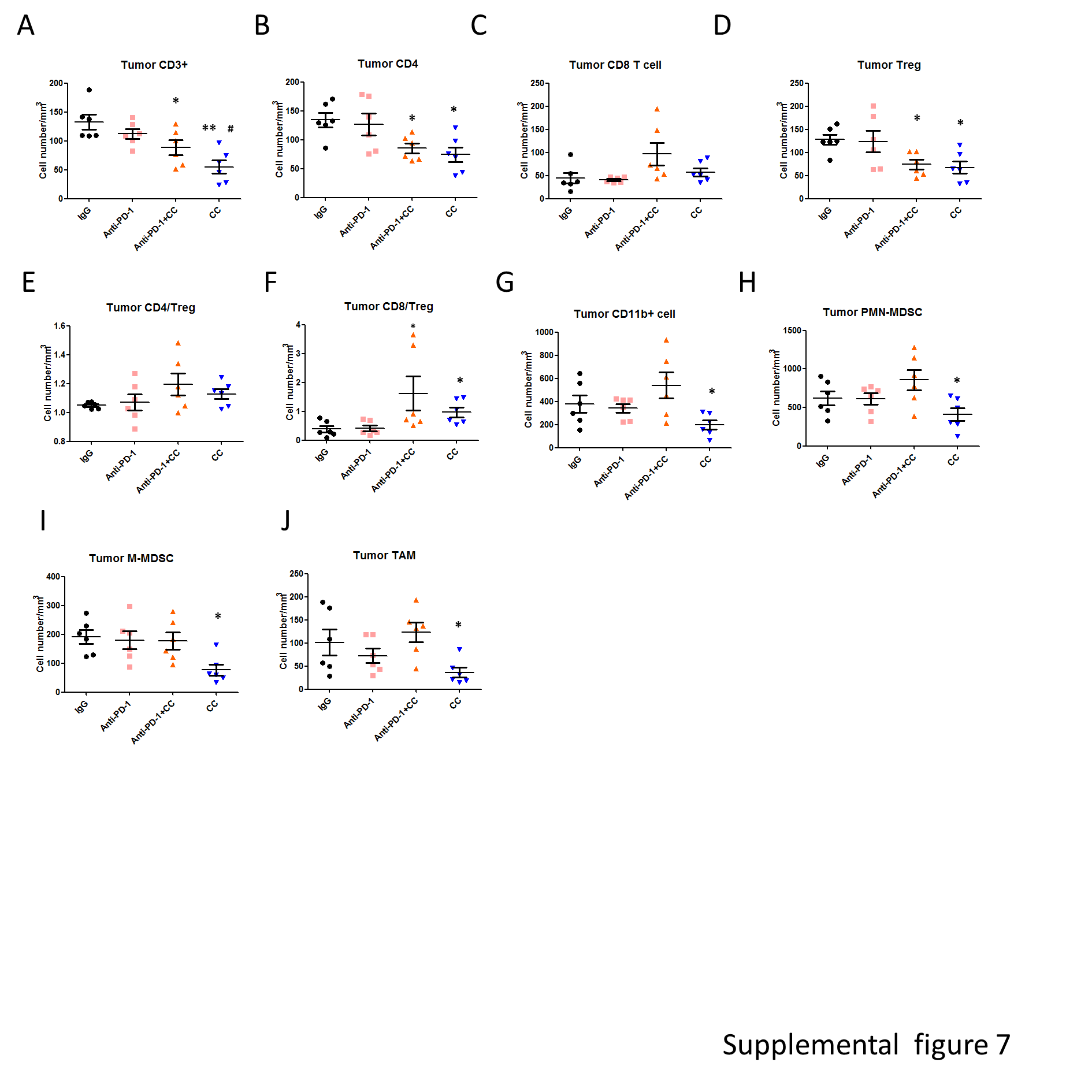


**S-Figure 7. Infiltrating immune cell populations of lymphocytes and myeloid-derived MDSCs in tumors**

CT26 tumor-bearing mice were orally administered a chidamide-k30 solution and celecoxib (50 mg/kg) once daily from days 10 to 22 (Day 10 mean tumor volume (TV), 220-240 mm^3^). Tumor-infiltrating lymphocytes were isolated by the Percoll gradient centrifugation method. Results are shown as mean + SD. Using % of CD45^+^ X 10000 events (CD45^+^cells counts)/tumor volume to calibrate. **A-D.** Number of total T cells/mm3 tumor. Percentages of CD3^+^ , CD4^+^, CD8^+^ T cells and Tregs in tumors by flow cytometric analysis. **E and F.** CD4/Treg and CD8/Treg ratios in tumors by flow cytometric analysis. **G.** Flow cytometric analysis of myeloid-derived CD11b^+^ cells in tumors. **H and I.** Flow cytometric analysis of myeloid-derived PMN-MDSC and M-MDSC cell populations in tumors. **J.** Flow cytometric analysis of myeloid-derived Ly6C^+^/MHCll^+^ tumor-associated macrophages in tumors. Results are shown as mean +SD. *p<0.05 vs. anti-IgG. *p<0.05 or **p<0.01 vs. anti-IgG; #p<0.05 vs. anti–PD-1.


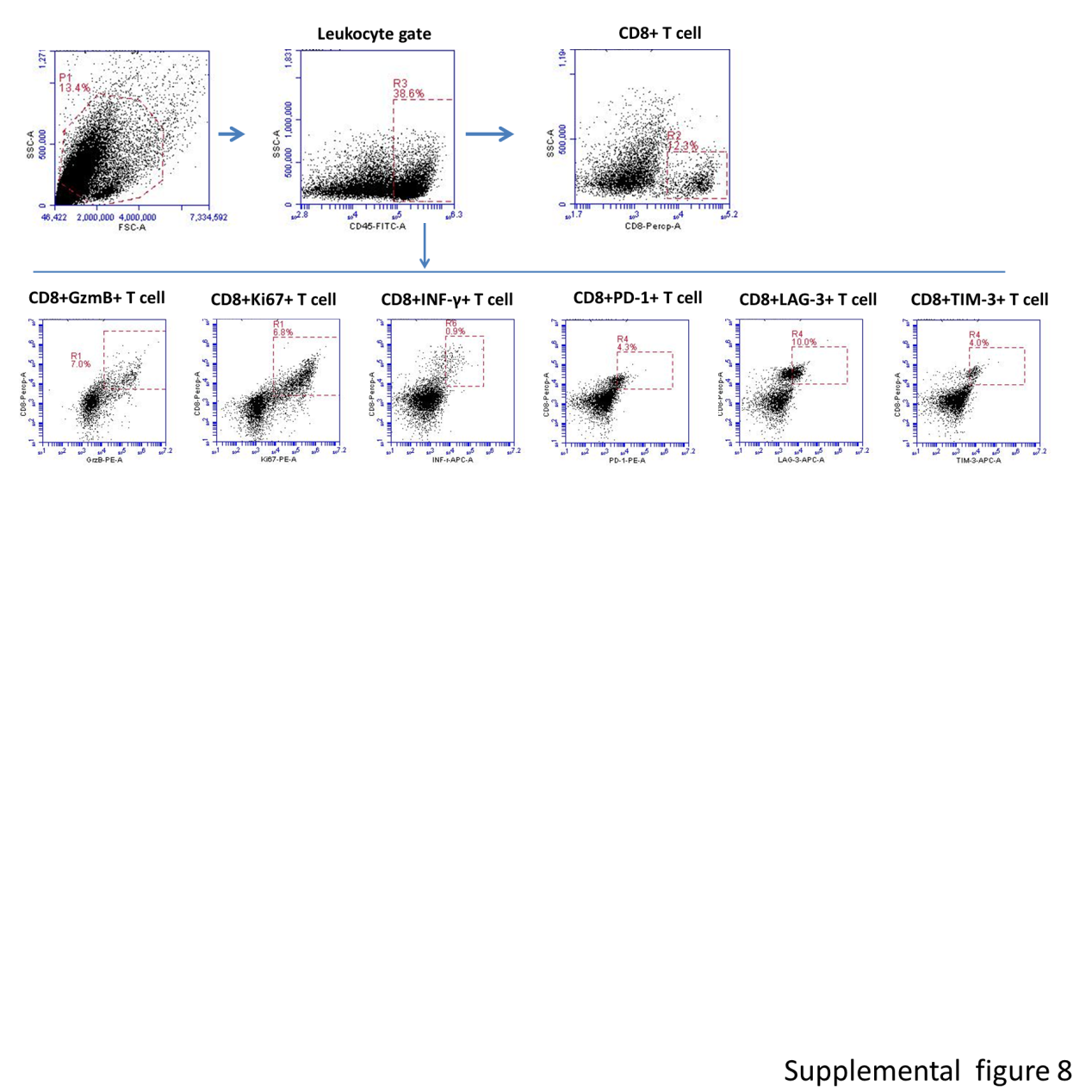


**S-Figure 8. Flow cytometric analysis of tumor infiltration of CD8^+^ T cells in BALB/c mice bearing CT26 tumors after treatment**

During flow cytometric subsets assay with gating strategy for leukocyte (CD45^+^), the level of tumor-infiltrating lymphocytes in mice tumor was showed as cell number per gram tumor weight. The leukocytes were first purified from tumor samples excised on day 12 after initial treatment of chidamide + celecoxib, with (represented in the Figure) or without anti–PD-1 antibody, and separated by Percoll gradient to isolate mononuclear cells. Flow cytometry analysis of CD8^+^ T cell was stained for the T cell marker (CD45^+^/ CD8^+^) and showed as the percentage of leukocyte. Flow cytometry analysis of CD8^+^GzmB^+^ cell (CD45^+^/CD8^+^/GzmB^+^), CD8^+^Ki67^+^ cell (CD45^+^/CD8^+^/Ki67^+^), CD8^+^INF-γ^+^ cell (CD45^+^/CD8^+^/INF-γ^+^), CD8^+^LAG-3^+^ cell (CD45^+^/CD8^+^/LAG-3^+^), CD8^+^PD-1^+^ cell (CD45^+^/CD8^+^/PD-1^+^), and CD8^+^TIM-3^+^ cell (CD45^+^/CD8^+^/TIM-3^+^) were showed as the percentage of CD8^+^ T cell. Representative dot plot is shown.


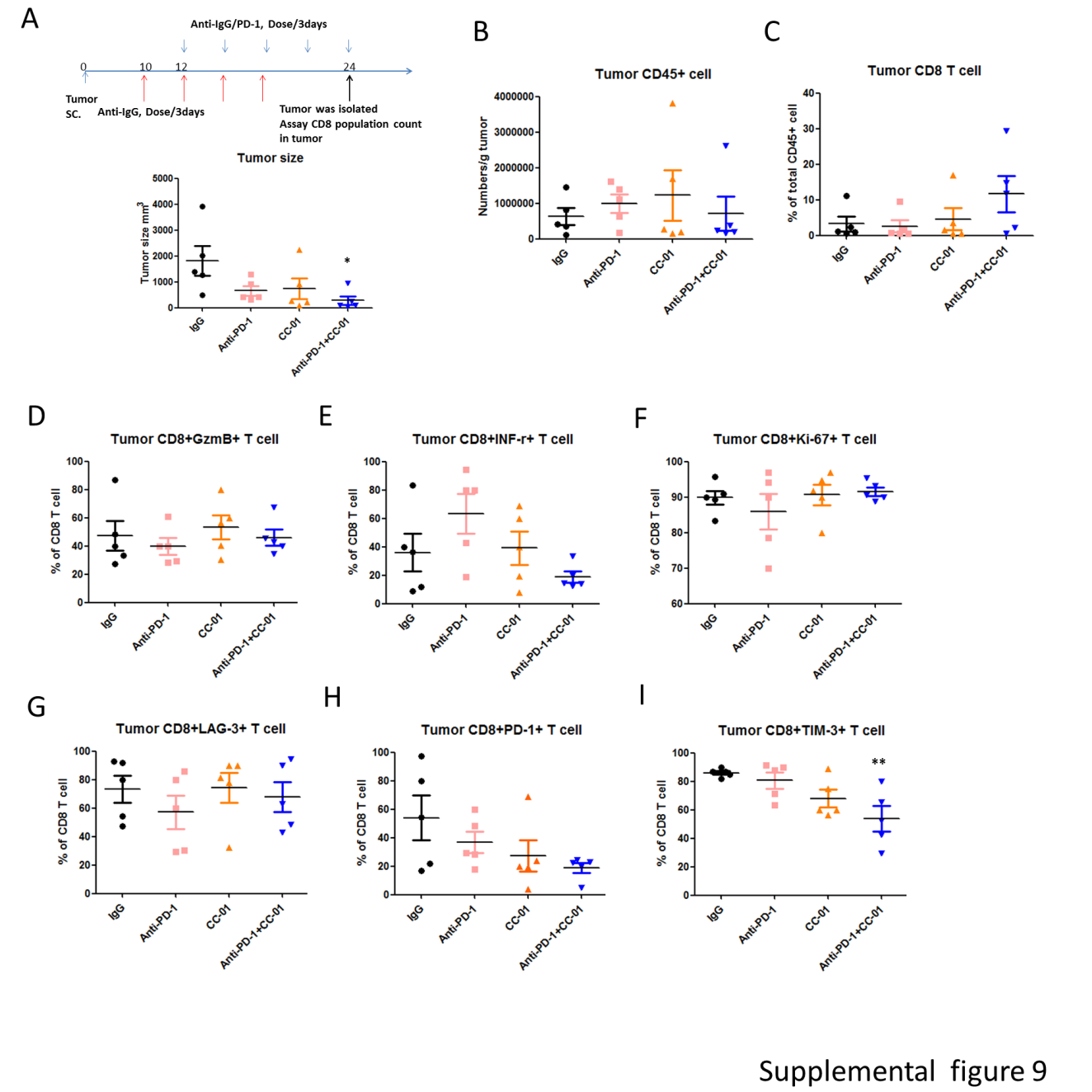


**S-Figure 9. Flow cytometric analysis of tumor infiltration of CD8^+^ T cells in BALB/c mice bearing CT26 tumors for each treatment group**

**A.** The treatment schedule; **B.** The cell numbers of leukocytes in CT26 tumors; **C.** Percentages of CD8^+^ in leukocyte population; **D.** GzmB^+^ CD8^+^ T cells ; **E.** IFN-γ^+^ CD8^+^ T cells; **F.** Ki67^+^ CD8^+^ T cells; **G.** LAG-3^+^ CD8^+^ T cells; **H.** PD-1^+^ CD8^+^ T cells; and **I.** TIM-3^+^CD8^+^T cells. The gating strategy is shown in S-Figure 6. *P <0.05, **P <0.01, Dunnett’s test vs. vehicle (n = 5).

**
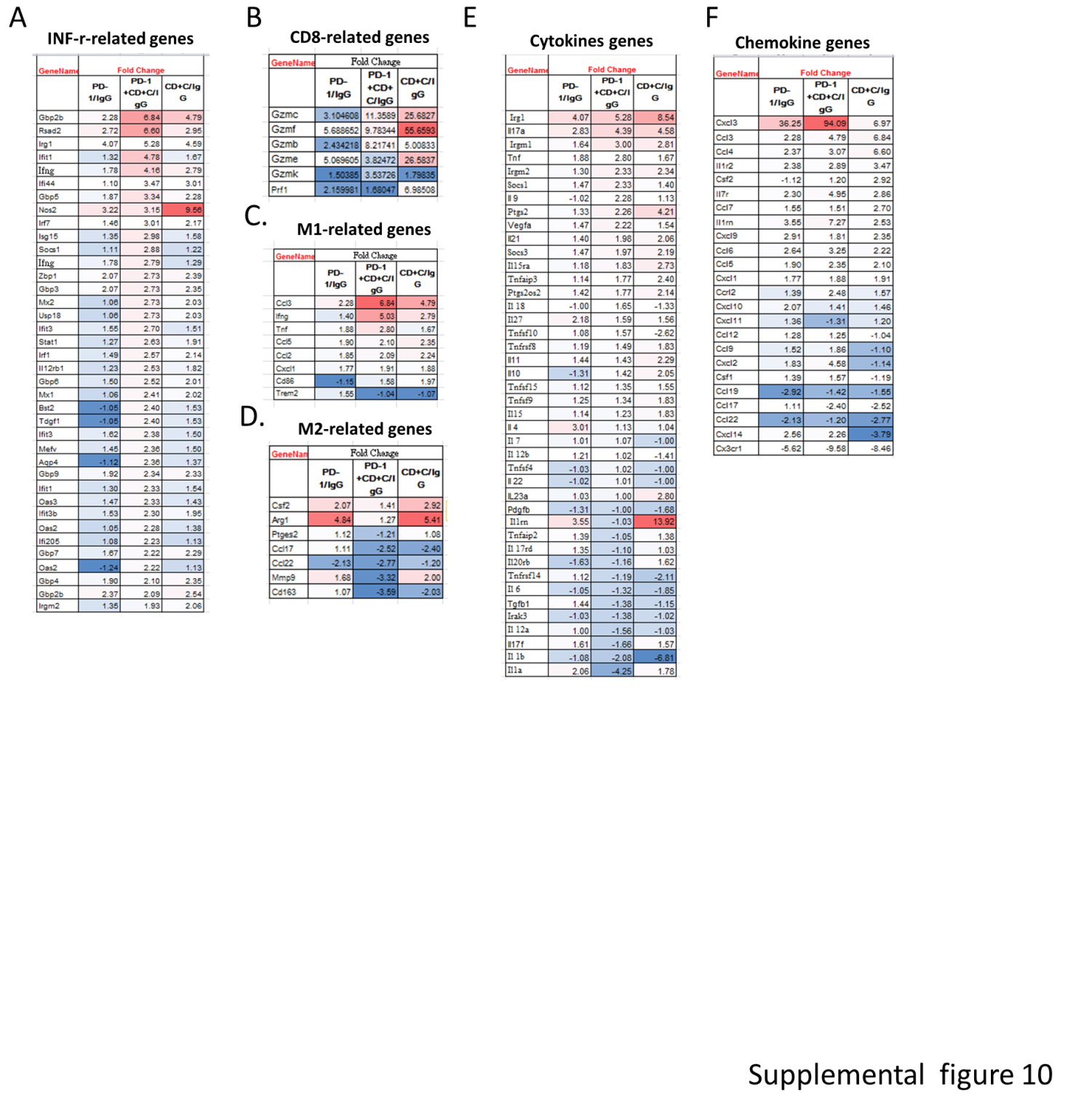
**

**S-Figure 10. CT26 tumor tissue analyses revealed chidamide + celecoxib-triggered up-regulation or down-regulation of inflammatory response-related gene expression using DNA microarray chip.** CT26 tumor-bearing mice were orally administered a chidamide-k30 solution (CD) and celecoxib (C, 50 mg/kg) once daily from days 10 to 22 (Day 10 mean tumor volume (TV), 220-240 mm^3^). The mice were treated with anti–PD-1 antibody (2.5 mg/kg, i.p.) once every 3 days on days 10, 13, 16, 19, and 22. The tumor was isolated on day 12 after initial treatment. Differentially expressed genes in the CT-26 tumor in response to the indicated treatment are listed. Heat map representation depicting the induced- and reduced-expression of inflammatory-related genes in the tumor tissue on day 12 after indicated treatment ( log2-fold change ≥ 1.0 or < 0.5). Gene expression levels were compared between tumors treated with anti–PD-1, anti–PD-1+CD+C, CD+C vs. IgG-treated cells. The Z-score for the mean expression level of each gene is depicted according to the color scale. The functional categorization of significant upregulation and downregulation of genes induced by any of the treatment (anti–PD-1, anti–PD-1+CD+C or CD+C) (with a fold change of >2 or <0.5), are shown in the figures. **A.** The regulated genes involved in IFN-γ-related immune response. **B.** The regulated genes related to CD8 T cell activation. **C.** The regulated genes involved in the activation of M1 macrophages. **D.** The regulated genes involved in the activation of M2 macrophage. **E and F.** The regulated genes related to thechemokines and cytokines.


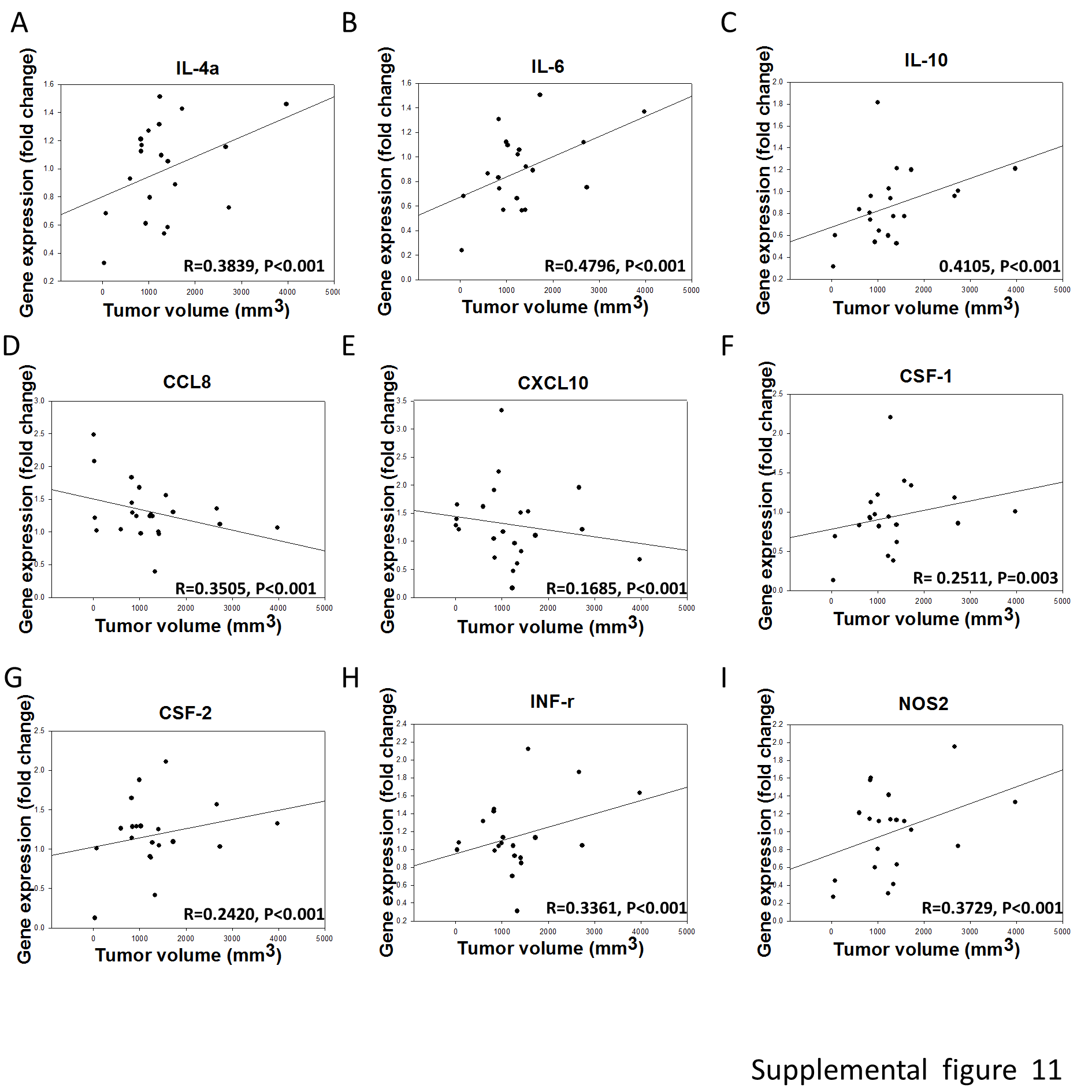


**S-Figure 11. Assay for the correlation between the chemokine expression level and tumor size of allograft tumors following indicated treatment**. Total mRNA was extracted from all treated tumors (n = 22 for each group), and mRNA levels of genes were determined by qPCR. **A.** IL-4a is positively correlated with tumor size; **B.** IL-6 is positively correlated with tumor size; **C.** IL-10 is positively correlated with tumor size; **D.** CCL8 is negatively correlated with tumor size; **E.** CXCL10 is negatively correlated with tumor size; **F.** CSF-1 is positively correlated with tumor size; **G.** CSF-2 is positively correlated with tumor size; **H**. INF-γis positively correlated with tumor size due to higher gene expression from two large tumors of the Ig G control group; **I.** NOS2 is positively correlated with tumor size.
